# The role of microRNAs and long non-coding RNAs in epigenetic regulation of T cells: implications for autoimmunity

**DOI:** 10.3389/fimmu.2025.1695894

**Published:** 2025-11-25

**Authors:** Soumil Prasad, Harini Adivikolanu, Abhinav Banerjee, Mannat Mittal, Joana R. N. Lemos, Rahul Mittal, Khemraj Hirani

**Affiliations:** 1 Diabetes Research Institute, University of Miami Miller School of Medicine, Miami, FL, United States; 2 Division of Endocrinology, Diabetes, and Metabolism, Department of Medicine, University of Miami Miller School of Medicine, Miami, FL, United States

**Keywords:** T cells, autoimmunity, epigenetics, microRNA, long non-coding RNA, type 1 diabetes

## Abstract

Non-coding RNAs (ncRNAs), comprising microRNAs (miRNAs) and long non-coding RNAs (lncRNAs), are increasingly recognized as central regulators of epigenetic programming in T lymphocytes with critical implications for immune tolerance and autoimmunity. We conducted a systematic review to investigate the influence of non-coding RNAs on DNA methylation, histone modifications, and chromatin accessibility in T cells across diverse autoimmune diseases. The majority of studies identified consistent patterns of dysregulation, including increased expression of miR21, miR148a, and miR155, and decreased expression of miR146a, GAS5, and IL21AS1. These alterations were associated with hypomethylation of proinflammatory gene loci, reduction of repressive histone marks, and increased chromatin accessibility at promoters of genes driving pathogenic T cell responses. Mechanistic data from both human and animal models demonstrated that microRNAs frequently regulate the abundance or activity of DNA methyltransferases and upstream signaling molecules, whereas long non-coding RNAs influence the recruitment or activity of chromatin modifying complexes, serve as scaffolds for transcriptional regulators, or function as competitive endogenous RNAs. Experimental manipulation of these non-coding RNAs attenuated disease-associated epigenetic and functional changes in T cells, supporting a causal role in autoimmune pathogenesis. Collectively, the non-coding RNAs as potential biomarkers of disease activity and as therapeutic targets capable of restoring physiological epigenetic regulation in a cell type specific manner. Future research should prioritize longitudinal and single cell multiomics approaches to delineate the dynamic interactions between non-coding RNAs and the chromatin landscape in order to accelerate the translation of these findings into targeted RNA based interventions for autoimmune diseases.

**Systematic review registration:**

https://inplasy.com/, Identifier INPLASY202580041.

## Introduction

T cells play a central role in adaptive immunity, and their dysfunction is one of the key drivers of autoimmune disorders (1). Proper T cell differentiation and function depend on tightly regulated gene expression programs, which are in turn controlled by epigenetic mechanisms such as DNA methylation and histone modifications (2, 3). Aberrant epigenetic modifications in T cells have been linked to the loss of self-tolerance; for example, global DNA hypomethylation and altered histone marks in T cells are observed in systemic lupus erythematosus (SLE), rheumatoid arthritis (RA), Type 1 diabetes (T1D) and other autoimmune disorders (2, 4). These findings suggest that epigenetic regulation is indispensable for maintaining T cell homeostasis, and its disruption can contribute to autoimmunity.

Epigenetic changes in T cells can be influenced by environmental and genetic factors (5). Environmental exposures such as chemicals, diet, and inflammation can trigger abnormal DNA methylation or histone modification patterns in CD4+ T cells, leading to deregulation of genes that determine T cell identity and function (5–10). Notably, genes like *IFNG*, *CD70*, *TNF*, and *FOXP3*, which are critical for effector functions or regulatory T cell (Treg) development, have been shown to undergo epigenetic dysregulation in autoimmunity in response to external triggers (11–13). Among the epigenetic regulators, non-coding RNAs have emerged as particularly important mediators of these environmentally induced changes (14). Indeed, recent advances in immunology have documented that microRNAs and long non-coding RNAs (lncRNAs) are integral components of the immune epigenetic landscape, acting as fine-tuners of gene expression in T cells.

**Figure 1:**
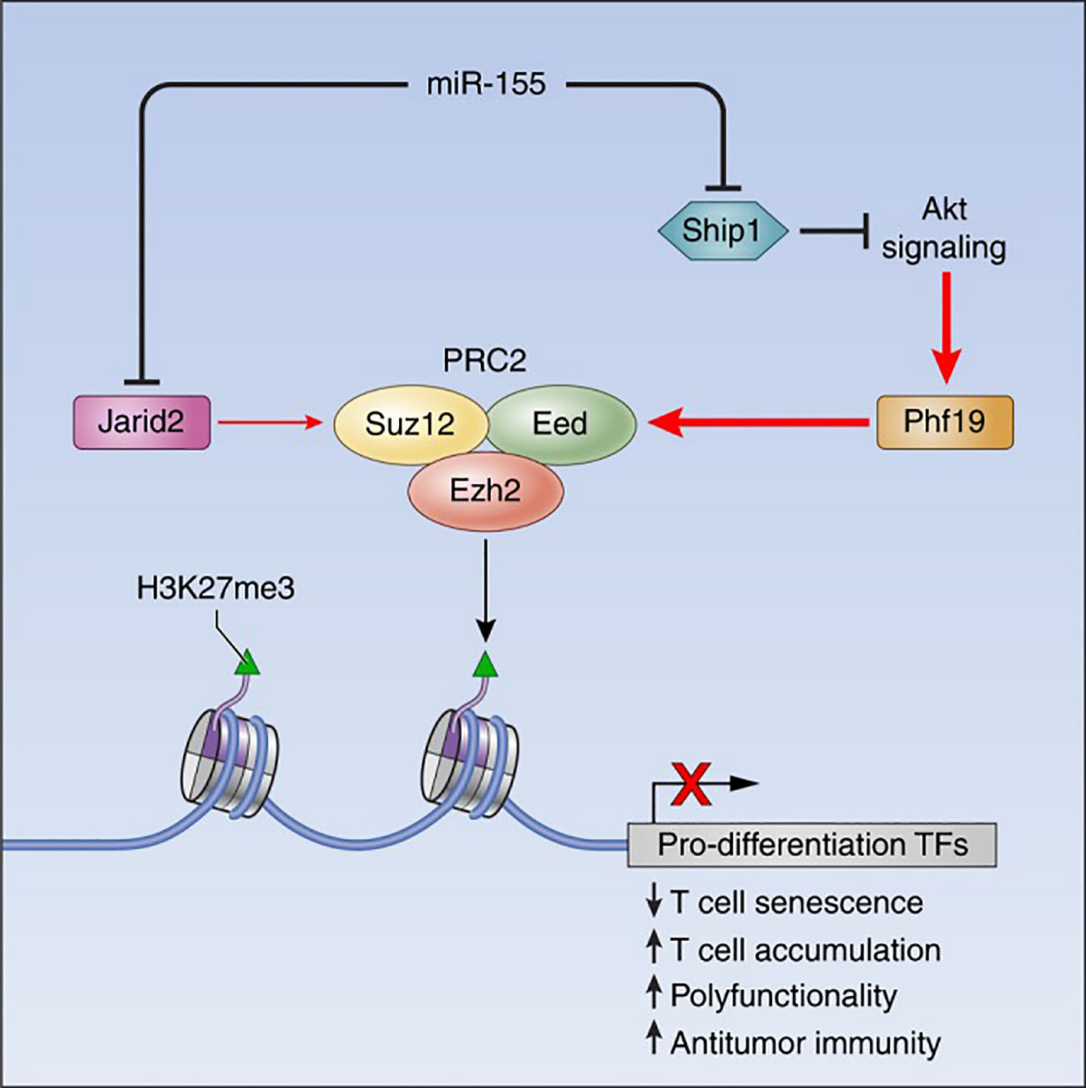
The miR-155-PRC2 circuitry potentiates antitumor immunity by epigenetically reprogramming CD8^+^ T cell fate. A schematic representation depicting the regulatory circuitry by which miR-155 epigenetically reprograms CD8^+^ T cell fate and function via enhancement of PRC2 activity. TFs, Transcription factors. Taken from (17) under a Creative Commons Attribution 4.0 International License.

MicroRNAs (miRNAs) are ~22-nucleotide non-coding RNAs that post-transcriptionally repress target gene expression (15). They are critical for immune cell development and tolerance, as they provide a layer of posttranscriptional epigenetic regulation (regulation of gene output without altering DNA sequence) (16). Dysregulation of miRNAs can permit the survival or activation of autoreactive lymphocytes, breaching self-tolerance. Seminal studies in both mice and humans have shown that miRNAs orchestrate key signaling pathways (such as PI3K–AKT, and NF-κB) that govern T cell selection, survival, and differentiation. **Figure 1** provides an example of such regulation, illustrating how miR-155 interacts with the Polycomb repressive complex 2 (PRC2) to epigenetically reprogram T cell fate. This circuitry highlights how a single miRNA can influence chromatin states and drive long-term changes in immune cell function (17). In autoimmune diseases such as SLE, RA, and multiple sclerosis (MS), distinctive miRNA expression patterns have been observed in T cells. For instance, overexpression of certain miRNAs in lupus CD4+ T cells (notably miR-21, miR-148a, and miR-126) directly targets the DNA methylation machinery, leading to reduced DNA methyltransferase 1 (DNMT1) levels and global DNA hypomethylation (18). This loss of methylation in turn causes aberrant overexpression of genes that promote autoreactive T cell behavior (18). Thus, miRNAs can act as epigenetic rheostats in T cells, and their aberrant activity is a plausible driver of autoimmune pathology.

**Figure 2:**
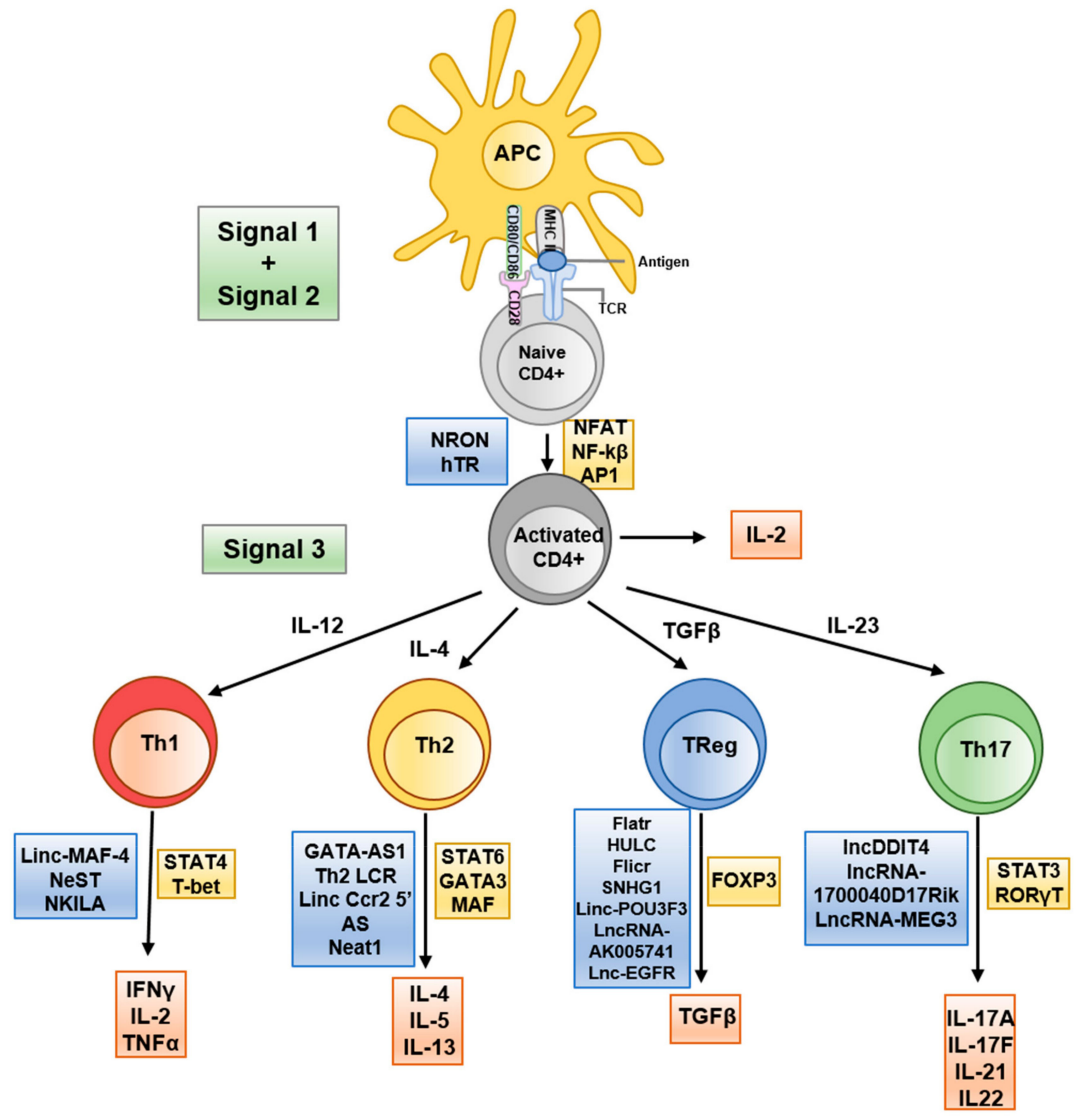
Schematic representation of T helper differentiation and associated lncRNA. Key secreted cytokines (red boxes), transcribed transcription factors (yellow boxes) and implicated lncRNAs (blue boxes) are indicated at the appropriate transitional stages. Taken from (20) under the terms and conditions of the Creative Commons Attribution (CC BY) license.

Long non-coding RNAs (lncRNAs), defined as transcripts >200 nucleotides with no protein-coding function, have recently been recognized as versatile regulators of gene expression in the immune system (19). LncRNAs often function by interacting with chromatin, transcription factors, or other RNAs to modulate gene expression at the epigenetic and transcriptional level (**Figure 2**). Immune cells express thousands of distinct lncRNAs in a cell type- and activation-specific manner. Although the majority of these remain uncharacterized, a growing subset has been shown to exert immune regulatory functions. In T cells, lncRNAs can guide epigenetic modifiers to specific gene loci or act as “sponges” for miRNAs, thereby influencing chromatin accessibility and gene transcription programs (**Figure 2**) (20). For example, the lncRNA Flicr (Foxp3 lineage-associated non-coding RNA) is transcribed near the *FOXP3* gene and has been shown to reduce chromatin accessibility at the FOXP3 locus, leading to lower FOXP3 expression and weakened Treg differentiation (21). Mice lacking or dysregulating Flicr exhibit increased susceptibility to autoimmune diabetes due to impaired Treg function (22). Another lncRNA, MALAT1, is broadly expressed in immune cells and has garnered attention for orchestrating epigenetic and transcriptional mechanisms in autoimmunity (23). MALAT1 influences a wide spectrum of cellular processes, including cell differentiation, apoptosis, and cytokine production, partly by modulating epigenetic modifications and by interacting with other RNAs and chromatin-associated proteins (23). Dysregulated MALAT1 expression is observed across several autoimmune diseases, often correlating with disease severity, highlighting its potential importance in immune pathogenesis (24–26).

Aberrant expression of miRNAs and lncRNAs in T cells has been observed in various autoimmune conditions, including SLE, RA, MS, psoriasis, and T1D. These ncRNAs collectively contribute to an “epigenetic signature” of autoimmunity. For instance, SLE patients’ CD4+ T cells display both abnormal miRNA profiles (such as high miR-155, miR-21, miR-148a, low miR-146a) and lncRNA profiles (low GAS5, linc0597) that distinguish them from healthy T cells (27). Such changes are not merely consequences of disease; rather, many studies suggest they actively drive pathogenic T cell phenotypes. In murine models, deletion of a single miRNA can precipitate or prevent autoimmune disease by altering T cell tolerance checkpoints. Likewise, overexpression or knockdown of specific lncRNAs in T cells can modulate autoimmunity severity by epigenetically reprogramming T cell responses (28). These observations point to a unifying concept: ncRNAs are key endogenous regulators of T cell epigenetic states, and their dysregulation can tip the balance between tolerance and autoimmunity.

Despite growing evidence for individual ncRNA roles, there has not yet been a comprehensive synthesis of how miRNAs and lncRNAs together contribute to epigenetic regulation in T cells across different autoimmune diseases. Questions remain as to which epigenetic mechanisms are commonly targeted by ncRNAs in T cells, how findings from animal models translate to humans, and whether these insights can be harnessed for therapeutic benefit. Here, we present a systematic review of the literature to address these gaps. We focus on primary research studies that investigate miRNAs or lncRNAs in relation to T cell epigenetic modifications (such as DNA methylation, histone modifications, and chromatin accessibility), with a special emphasis on autoimmune disease contexts. By analyzing findings across diverse study types (clinical observational studies, *in vitro* experiments, and animal models), we aim to identify recurring mechanistic themes and evaluate the potential for translational applications. Understanding the integrated role of ncRNAs in T cell epigenetic regulation will not only illuminate fundamental disease mechanisms but also pave the way for innovative RNA-targeted therapies for autoimmune disorders. This review is timely given the surge of interest in therapeutic modulation of the epigenome and the advent of RNA-based drugs; it consolidates current knowledge and points toward future research needed to translate these insights into clinical advances.

## Methods

### Study design

This systematic review was conducted in accordance with the Preferred Reporting Items for Systematic Reviews and Meta-Analyses (PRISMA) 2020 guidelines (29). This systematic review was designed *a priori* and registered in the INPLASY database prior to the commencement of this study (registration number: INPLASY202580041).

### Search strategy

A comprehensive literature search was performed to identify studies on microRNAs or long non-coding RNAs involved in epigenetic regulation of T cells, particularly in the context of autoimmunity and immune-mediated disease. We queried MEDLINE (via PubMed), The search strategy combined controlled vocabulary and free-text terms for three key concepts: (1) T cells (*T lymphocyte*, *CD4* or *CD8 T cell*, and *lymphocyte*), (2) non-coding RNAs (*microRNA*, *miRNA*, *long non-coding RNA*, *lncRNA*, specific RNA names like *miR-146a*, and *MALAT1*), and (3) epigenetic regulation (such as *epigenetic*, *DNA methylation*, *histone modification*, *chromatin*, *DNMT*, and *histone acetylation*), along with terms for autoimmunity and related diseases (such as *autoimmune*, *autoimmunity*, *lupus*, *arthritis*, and *multiple sclerosis).* Searches were run without language limits in MEDLINE via PubMed from database inception to 1 March 2025. Reference lists of all eligible articles and relevant reviews were scanned manually to capture additional records.

All citations were exported to Covidence, which performed automatic de-duplication; out of 1002 records retrieved, seventy-three duplicates were removed, leaving 929 unique titles and abstracts. Two reviewers independently screened titles and abstracts against pre-specified criteria: original research that examined the influence of any class of non-coding RNA on epigenetic marks in primary or experimentally manipulated T cells from humans or murine models of immune-mediated disease. No language restrictions were applied, but we limited the results to peer-reviewed primary research articles (excluding reviews, commentaries, and conference abstracts). The reference lists of relevant review articles identified during the initial search were manually screened to identify additional studies meeting the eligibility criteria. All database search results were imported into Covidence for deduplication and screening. Reviews, editorials, case reports, studies focused exclusively on coding genes or on non-T-cell populations, and papers that lacked an epigenetic end-point were excluded. This process advanced 98 articles to full-text review, after which 52 were excluded for failing to meet inclusion criteria, leaving a final analytical dataset of 46 studies. Disagreements were resolved by discussion with a third reviewer.

Cohen’s κ statistic was utilized to determine inter-rater agreement (30, 31). Interpretation of κ values followed established thresholds: <0.00 denoted no agreement, 0.00–0.20 slight agreement, 0.21–0.40 fair agreement, 0.41–0.60 moderate agreement, 0.61–0.80 substantial agreement, and 0.81–1.000 almost perfect agreement. Consistent with prior literature, a κ value exceeding 0.60 was defined as indicative of substantial agreement, thereby providing sufficient reliability to advance to subsequent phases of the study (32–34).

### Study selection

Three reviewers (SP, HA and AB) independently screened the titles and abstracts yielded by the search to identify potentially relevant studies. Prior to screening, pilot screening exercises were conducted to ensure consistency between reviewers. After initial screening, the full texts of all studies deemed potentially eligible were obtained and assessed in detail against the inclusion/exclusion criteria. Discrepancies in study selection were resolved by discussion with senior authors (RM and KH) when necessary.

The inclusion criteria were as follows: (1) studies investigating one or more microRNAs or lncRNAs *in T cells* (human or animal) with respect to epigenetic regulation, i.e., studies that measured the effect of ncRNAs on epigenetic marks (DNA methylation, histone modifications, chromatin state) or vice versa; (2) studies focused on autoimmune diseases or related inflammatory disorders (such as SLE, RA, MS, T1D and psoriasis), including both human patient studies and relevant animal models; (3) primary research articles reporting original data (such as case-control, cross-sectional, intervention, or animal experimental studies); and (4) articles published in peer-reviewed journals. We excluded studies that did not specifically examine epigenetic outcomes (for example, papers that only profiled ncRNA expression in T cells without assessing any epigenetic effect), studies not involving T cells (such as focusing on B cells or other immune cells), purely *in vitro* studies using transformed cell lines without a direct link to an autoimmune context, and non-original studies (reviews, editorials, and case reports). Where multiple papers reported results from the same cohort or experiment, we included the most comprehensive report to avoid double-counting data.

### Data extraction and quality assessment

For each included study, three reviewers (SP, HA and AB) independently extracted relevant data using a standardized extraction form designed for this review. Extracted data included: study design (such as case-control, cross-sectional, and animal experiment), population and sample size (including disease context, patient demographics, or animal model details), type of non-coding RNA(s) investigated [specific miRNA(s) or lncRNA(s)], epigenetic outcomes measured (such as changes in DNA methylation levels, histone modification status, and gene-specific epigenetic changes), key results (such as differences in ncRNA expression between groups, correlations between ncRNA and epigenetic markers, effects of ncRNA manipulation on epigenetic or immune outcomes), and conclusions. When provided, we also recorded additional notes such as cytokine or gene expression changes, and any mechanistic experiments (such as reporter assays, and knockdown/overexpression experiments) that linked the ncRNA to epigenetic modifications. Data extraction was cross-verified by the reviewers, and any discrepancies were reconciled through discussion or discussion with the senior authors (RM and KH).

We assessed the risk of bias (methodological quality) of the included studies using tools appropriate to each study design. Specifically, for observational studies (case-control and cross-sectional studies, as well as observational cohort or uncontrolled before-after studies), we used the Joanna Briggs Institute (JBI) Critical Appraisal Checklists for each respective design (35–37). These checklists evaluate potential biases in areas such as selection of participants, measurement of exposures and outcomes, identification and control of confounding factors, and completeness of outcome data. For the quasi-experimental studies (such as non-randomized intervention studies or *in vitro* experiments using patient-derived T cells), we employed the JBI checklist for quasi-experimental studies (which addresses risk of bias in the absence of randomization, such as whether multiple measurements were taken pre- and post-intervention, and whether outcome assessment might be biased). For animal studies, we utilized the SYRCLE (Systematic Review Centre for Laboratory animal Experimentation) risk-of-bias tool, which is an adaptation of the Cochrane tool tailored to animal research (38–41). The SYRCLE tool examines biases specific to animal experiments, including selection bias (random housing, random outcome assessment), performance bias (blinding of investigators), detection bias (blinding of outcome assessment), attrition bias, and reporting bias. Each study was assessed by two independent reviewers for risk of bias; judgments were categorized as “Low risk,” “High risk,” or “Unclear risk” for each domain of the respective tool. We resolved any discrepancies in ratings through consensus. To summarize the quality assessments, we generated visual risk-of-bias summary figures by study type (see Results). We did not exclude studies based on quality, but the risk-of-bias findings were taken into account when interpreting the results.

### Data synthesis

Given the diversity of study designs and outcomes, a meta-analysis was not deemed appropriate. Instead, we conducted a qualitative synthesis of the findings. We grouped studies by broad design and context (human observational studies; human quasi-experimental studies; animal studies) and also by the type of ncRNA (miRNA-focused vs lncRNA-focused) for the narrative synthesis. We present summary tables to compare study characteristics and results. These tables aggregate the key data extracted for easy reference (such as listing each study’s disease context, ncRNAs examined, main epigenetic findings, and major conclusions). In the narrative, we highlight consistent patterns or discrepancies across studies, and we map out proposed mechanistic pathways supported by the evidence. We also integrate findings from animal models with those from human studies to provide a translational perspective.

## Results

### Study selection and overall characteristics

**Figure 3:**
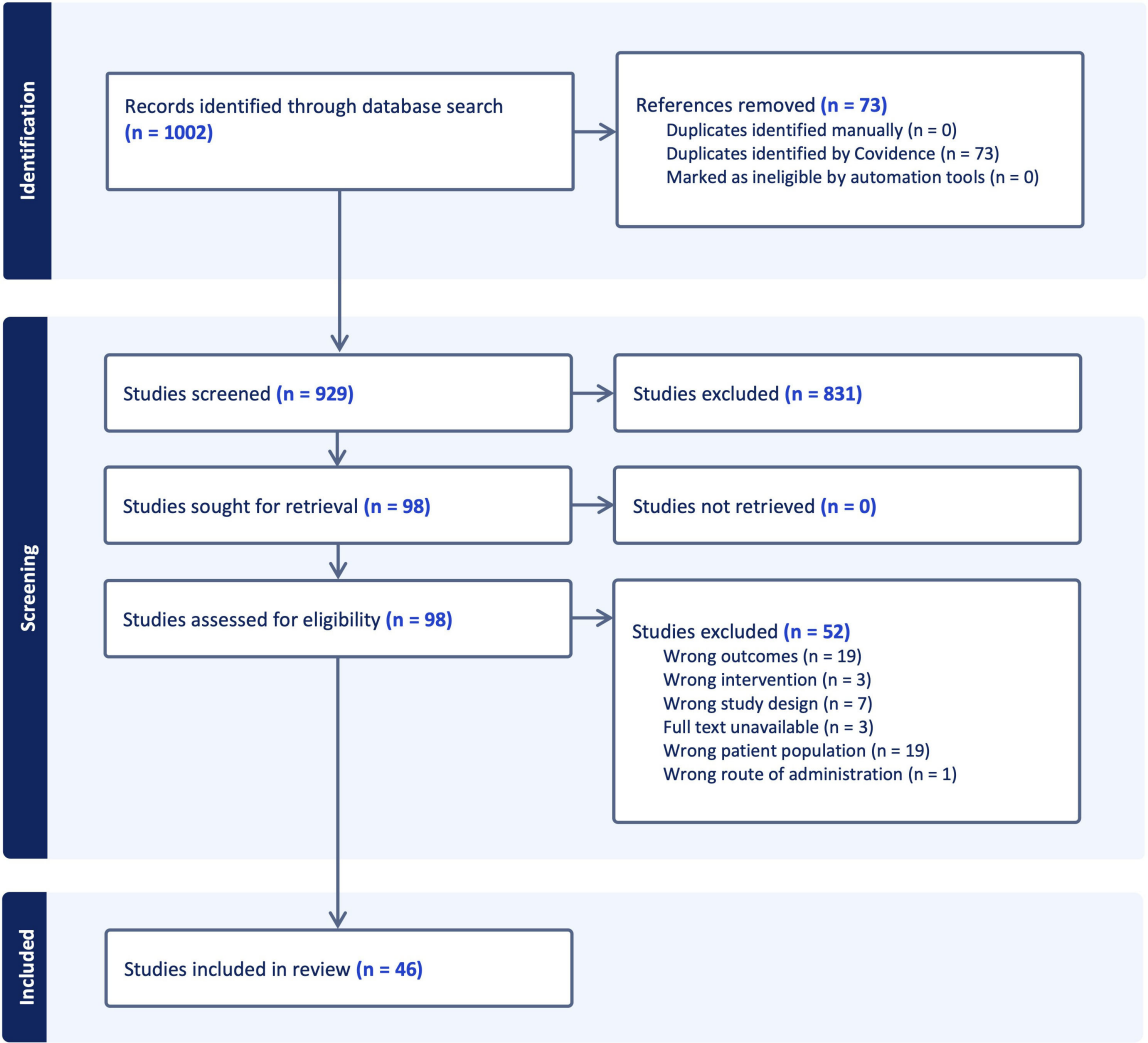
A PRISMA (Preferred Reporting Items for Systematic Reviews and Meta-Analyses) flow diagram of study selection process.

**Figure 4:**
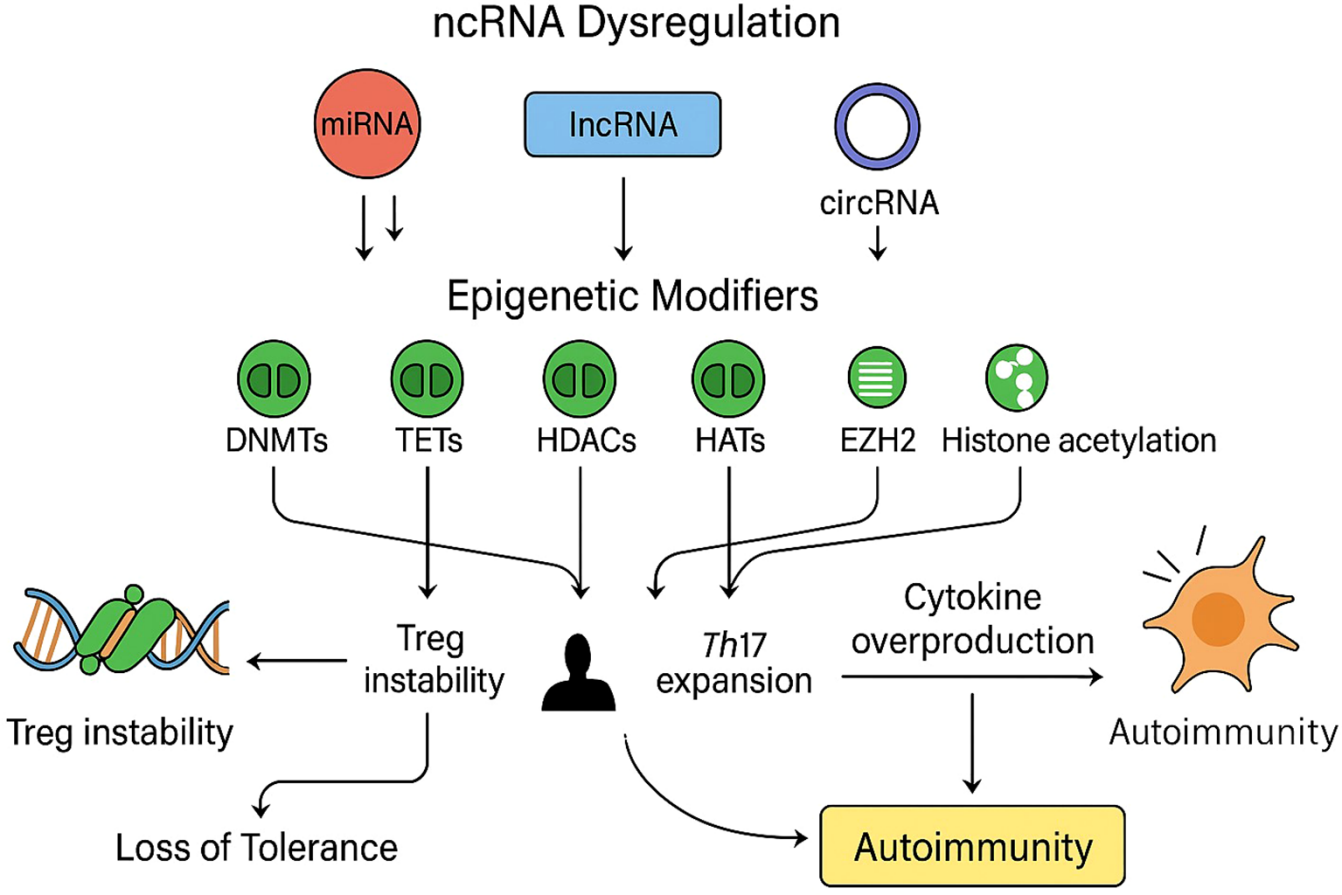
Non-coding RNA dysregulation and epigenetic effects in autoimmune T cells: A schematic representation of how dysregulated non-coding RNAs (ncRNAs), including microRNAs (miRNAs, red), long non-coding RNAs (lncRNAs, blue), and circular RNAs (circRNAs, purple), modulate key epigenetic regulators (green) such as DNA methyltransferases (DNMTs), ten-eleven translocation enzymes (TETs), histone deacetylases (HDACs), histone acetyltransferases (HATs), and enhancer of zeste homolog 2 (EZH2). Aberrant ncRNA expression disrupts normal epigenetic equilibrium by altering DNA methylation patterns, histone modification states, and chromatin accessibility, resulting in impaired regulatory T-cell (Treg) stability, enhanced T-helper 17 (Th17) differentiation, and excessive cytokine production. Collectively, these molecular alterations culminate in the breakdown of immune tolerance and the onset of autoimmune pathology.

**Figure 5:**
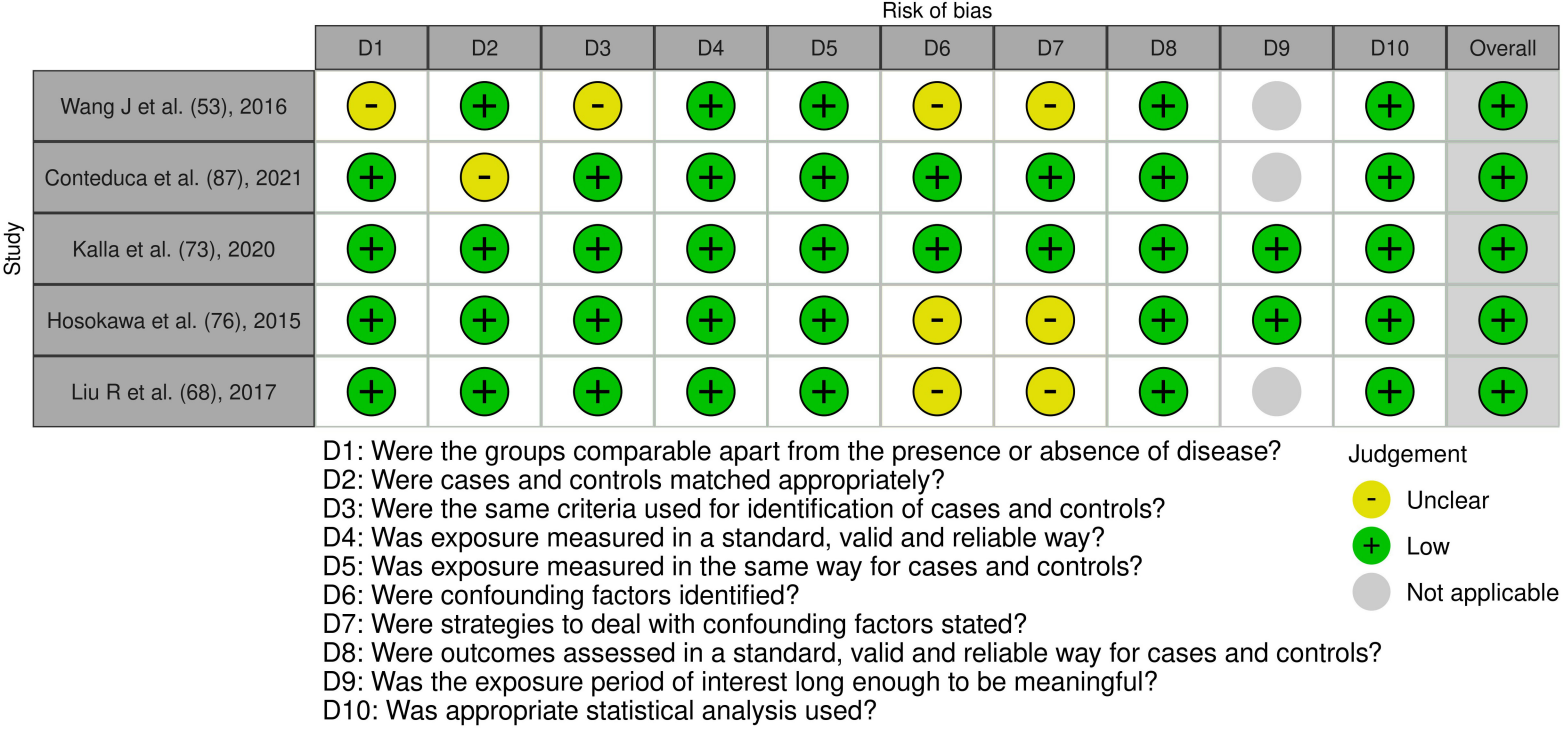
Risk of bias assessment of the included case control studies: This figure presents the risk of bias assessment of the included studies based on the Joanna Briggs Institute (JBI) Critical Appraisal Checklist for Case Control Studies. The color coding indicates the level of bias: green indicates low risk of bias, yellow indicates unclear risk, and gray indicates that the domain was not applicable.

**Figure 6:**
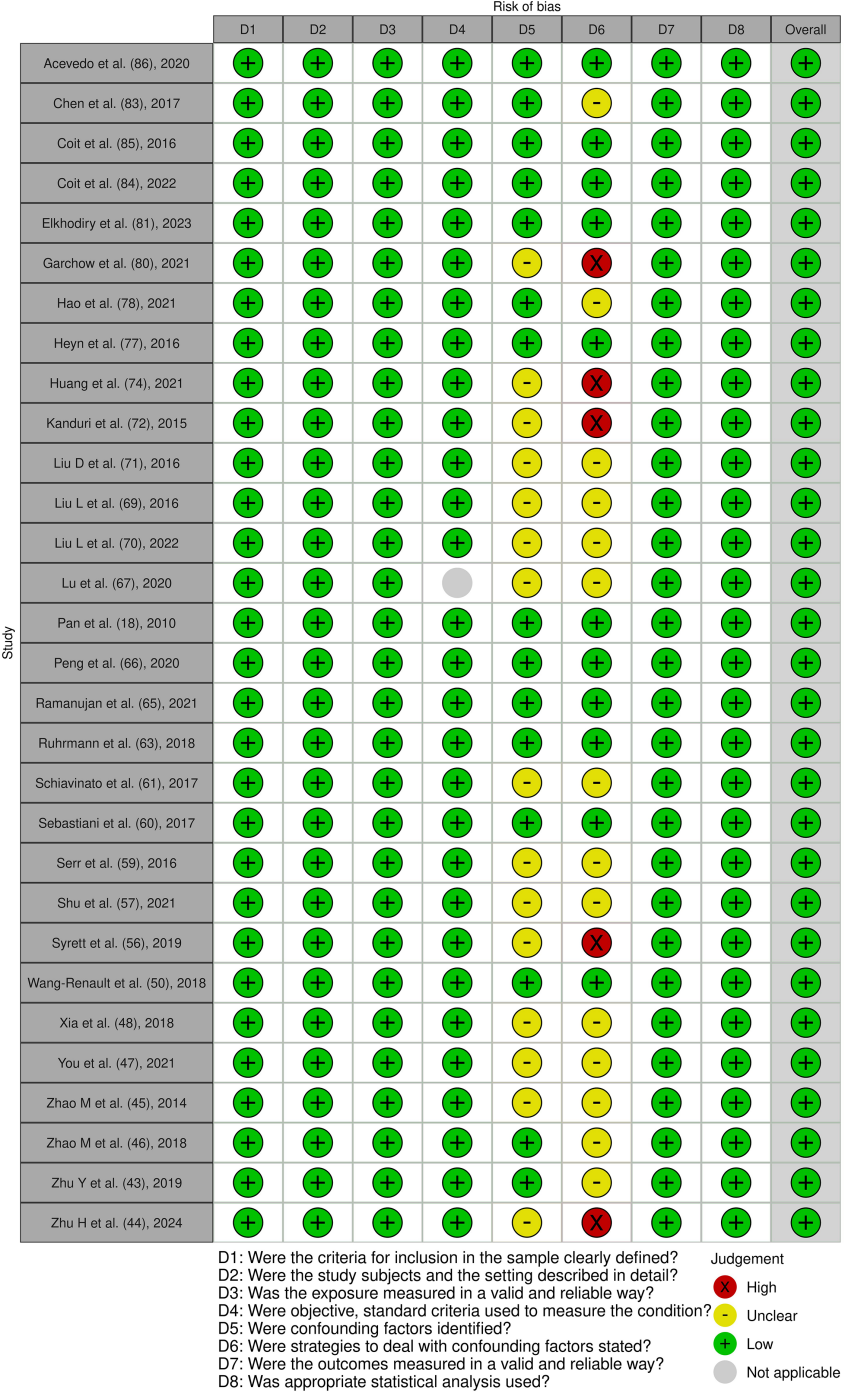
Risk of bias assessment of the included cross-sectional studies: This figure presents the risk of bias assessment of the included studies based on the Joanna Briggs Institute (JBI) Critical Appraisal Checklist for Quasi-Experimental Studies. The color coding indicates the level of bias: green indicates low risk of bias, yellow indicates unclear risk, red indicates high risk of bias, and gray indicates that the domain was not applicable.

**Figure 7:**
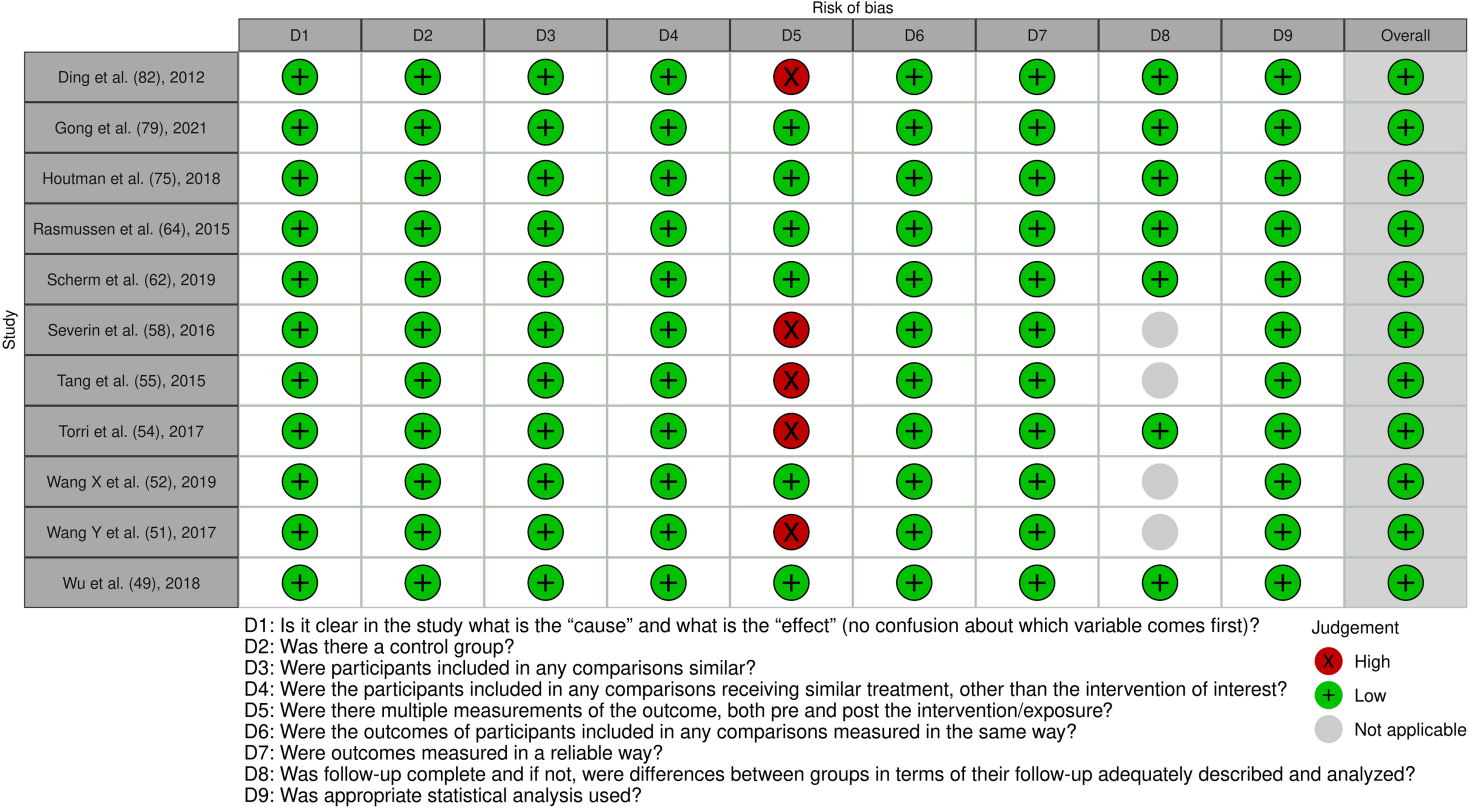
Risk of bias assessment of the included quasi-experimental studies: This figure presents the risk of bias assessment of the included studies based on the Joanna Briggs Institute (JBI) Critical Appraisal Checklist for Quasi-Experimental Studies. The color coding indicates the level of bias: green indicates low risk of bias, yellow indicates unclear risk, and red indicates high risk of bias.

**Figure 8:**
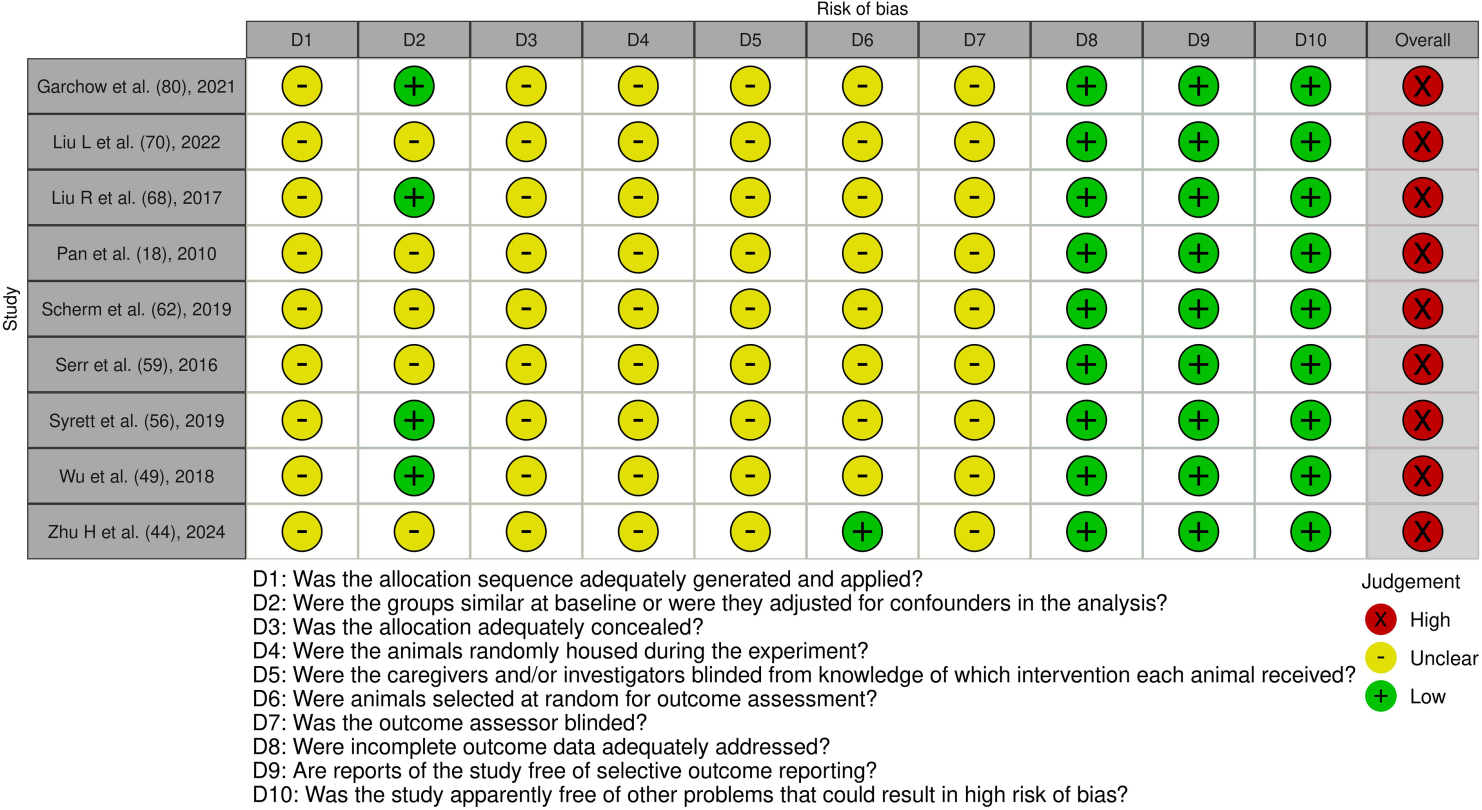
Risk of bias assessment of the included experimental animal studies: This figure presents the risk of bias assessment of the included studies with an experimental animal study component based on SYRCLE’s Risk of Bias tool for experimental animal studies. The color coding indicates the level of bias: green indicates low risk of bias, yellow indicates unclear risk, and red indicates high risk of bias.

Systematic screening identified 1002 unique citations; after removal of seventy-three duplicates, 929 titles and abstracts were assessed. Ninety-eight articles met criteria for full-text review and fifty-two were subsequently excluded, leaving forty-six studies for analysis. Thirty of the included investigations enrolled only human participants, seven employed animal models exclusively and nine combined human samples with *in-vivo* murine work. The study selection process is shown in the PRISMA flow diagram (**Figure 3**). A summary of included studies has been presented in **Supplementary Table 1**. A detailed summary of key microRNAs regulating the epigenetic landscape of T cells across autoimmune diseases is provided in **Table 1**. The overall patterns of ncRNA dysregulation and their epigenetic consequences are summarized in **Figure 4**. The risk of bias analysis for case control, cross-sectional and quasi-experimental study is shown in **Figures 5**–**7**. The risk of bias analysis for animal studies is shown in **Figure 8**.

**Table 1:** A summary of key microRNAs modulating the epigenetic architecture of T cells in autoimmunity.

microRNA	Validated target(s)	Epigenetic/molecular effect	Functional outcome in T Cells	Representative disease context(s)	References
miR-21	RASGRP1, DNMT1, BDH2	Indirect and direct suppression of DNMT1 leading to global DNA hypomethylation; altered hydroxymethylation via BDH2 repression	Promotes hyperactivation of CD4^+^ T cells; enhances pro-inflammatory gene expression	SLE, RA, MS	(18, 42–45)
miR-148a	DNMT1	Direct translational repression of DNMT1 causing DNA hypomethylation	Derepression of CD70 and LFA-1; drives autoreactive T cell activation	SLE	(18, 42, 45)
miR-155	SOCS1, PU.1	Modulates histone acetylation at IL17 and IFNG loci; promotes open chromatin configuration	Favors Th1/Th17 polarization; augments inflammatory cytokine production	MS, SLE, RA	(44, 46, 47)
miR-146a	IRAK1, STAT1, TRAF6	Dampens NF-κB signaling and downstream inflammatory gene transcription	Limits T cell activation; promotes immune tolerance	SLE, RA, ITP	(48–50)
miR-126	DNMT1	Inhibits DNMT1 leading to promoter hypomethylation of pro-inflammatory genes	Enhances CD70 and LFA-1 expression; contributes to autoreactivity	SLE	(42, 43, 45)
miR-142-3p	TET2, HDAC9	Suppresses TET2, reducing FOXP3 CNS2 demethylation and Treg stability	Impairs Treg differentiation; shifts Th17/Treg balance	T1D, SLE	(50, 51)
miR-92a	PTEN–Foxo1–KLF2 axis	Alters enhancer activity via Foxo1 signaling; promotes epigenetic activation of Tfh genes	Expands Tfh precursors; reduces Treg generation	T1D	(52)
miR-10a	Bcl-6, Sirt1	Increases histone acetylation at *FOXP3* locus	Stabilizes Tregs; suppresses effector T cell differentiation	RA, SLE	(53)
miR-210	HIF-1α (feedback loop)	Modulates chromatin accessibility at *RORγt* enhancer	Drives Th17 differentiation and IL-17 expression	SLE	(54)
miR-31	FOXP3	Induces FOXP3 promoter methylation and transcriptional repression	Reduces Treg differentiation; enhances effector T cell activity	Psoriasis	(55)

Bcl-6, B-cell lymphoma 6 protein; BDH2, 3-hydroxybutyrate dehydrogenase type 2; CD70, cluster of differentiation 70; CNS2, conserved non-coding sequence 2; DNMT1, DNA methyltransferase 1; FOXP3, forkhead box P3; Foxo1, forkhead box O1; HDAC9, histone deacetylase 9; HIF-1α, hypoxia-inducible factor 1 alpha; IRAK1, interleukin-1 receptor-associated kinase 1; ITP, immune thrombocytopenia; KLF2, Krüppel-like factor 2; LFA-1, lymphocyte function-associated antigen 1;miR, microRNA; MS, multiple sclerosis; PTEN, phosphatase and tensin homolog; PU.1, purine-rich box-1 transcription factor; RA, rheumatoid arthritis; RASGRP1, RAS guanyl releasing protein 1; RORγt, RAR-related orphan receptor gamma t;Sirt1, sirtuin 1; SLE, systemic lupus erythematosus; SOCS1, suppressor of cytokine signaling 1; STAT1, signal transducer and activator of transcription 1; TET2, ten-eleven translocation methylcytosine dioxygenase 2; Tfh, T follicular helper cell; Th1, T helper type 1; Th17, T helper type 17; T1D, type 1 diabetes; TRAF6, TNF receptor-associated factor 6; Treg, regulatory T cell.

Inter-rater agreement for study inclusion had a Cohen’s kappa of 0.81 indicating excellent consistency between reviewers during screening.

### Design categories

Among the forty-six studies, twenty-one used a cross-sectional analytical design, fifteen were case-control comparisons and ten incorporated an experimental or quasi-experimental component such as ex-vivo pharmacologic exposure, forced over-expression or genetic knock-down without randomization. Sixteen of the forty-six articles included *in-vivo* murine experiments, most often using MRL/lpr or NZB/W F1 lupus-prone strains, experimental autoimmune encephalomyelitis models for multiple sclerosis, NOD mice for T1D, or collagen-induced arthritis for rheumatoid arthritis. These studies extended mechanistic findings beyond primary human samples (4, 12–14, 18, 19, 23, 27, 29, 42–44, 46–81).

### Disease contexts studied

Systemic lupus erythematosus accounted for the largest share of papers (fifteen studies) and consistently centered on DNA hypomethylation or histone acetylation defects in CD4^+^ T cells (5, 11, 13, 18, 21, 27, 29, 43, 45, 48, 57, 59, 73, 74, 80). Multiple sclerosis, rheumatoid arthritis and T1D were each represented by three to five investigations (2, 14, 19, 28, 44, 47, 62–64, 82), whereas immune thrombocytopenia, ankylosing spondylitis, psoriasis, atopic dermatitis, primary Sjögren syndrome, inflammatory bowel disease, alopecia areata, Vogt-Koyanagi-Harada disease and acquired aplastic anemia were examined in one to three studies apiece (12, 23, 42, 46, 49, 50, 52, 55, 56, 60, 61, 65–69, 75–79, 81, 83–86). By leveraging parallel murine models, several groups confirmed that ncRNA-directed epigenetic shifts observed in patients also drive disease-relevant phenotypes *in vivo* (4, 51, 53, 54, 58, 70–72).

### Spectrum of non-coding RNAs interrogated

MicroRNAs dominated the field, featuring in thirty-four studies; miR-146a, miR-155, miR-21, miR-148a, miR-125b, miR-142-3p/5p and members of the miR-17~92 cluster were most frequently analyzed (2, 5, 11, 12, 18, 19, 21, 27–29, 43–50, 55–57, 59, 61–63, 65–69, 74, 81–83). Long non-coding RNAs were the primary focus in ten studies, including IL21-AS1, AC007278.2, IFNG-AS1, LINC01882 and the X-chromosome transcripts XIST and Flicr (4, 13, 42, 52, 58, 60, 64, 70, 73, 80), whereas three reports concentrated on circular RNAs such as circ_003912 in erosive oral lichen planus or mixed circRNA/miRNA signatures in Vogt-Koyanagi-Harada disease (23, 53, 77). A small subset examined interactions between lncRNAs and miRNAs, revealing competitive endogenous RNA networks that ultimately remodel chromatin (13, 52, 73, 80).

### Epigenetic end-points and mechanistic read-outs

All studies quantified at least one epigenetic outcome in T cells. Thirty-one papers measured DNA methylation, either globally or at specific promoters such as CD70, FOXP3, IL13 or MIR21, typically by bisulphite sequencing, Illumina 450 K arrays or MeDIP-qPCR (5, 18, 42, 43, 45, 62, 63, 69, 83, 84). Histone modifications were documented in eighteen studies, with particular emphasis on H3K27 trimethylation, H3K4 trimethylation and H3 acetylation at cytokine loci or ncRNA promoters; chromatin immunoprecipitation and ChIP-qPCR were the principal techniques, while one study incorporated ChIP-seq and another employed ATAC-seq to map genome-wide accessibility after ncRNA perturbation (11, 13, 28, 51, 80, 84). Seven investigations quantified chromatin-modifying enzymes directly, noting, for example, ncRNA-driven suppression of DNMT1, EZH2, TET2 or recruitment of the co-activator CBP (18, 28, 43, 45, 51, 80, 82). Functional correlates were routinely assessed through flow-cytometric detection of surface markers, western blots for signaling mediators and ELISA or intracellular staining for cytokines such as IL-17, IL-21, IFN-γ and IL-10, thereby linking epigenetic change to T-cell phenotype (21, 23, 44, 49, 58, 61, 66, 72).

### Findings of included studies

Non-coding RNAs and DNA methylation in T cells: A central theme across many studies was that dysregulated ncRNAs in autoimmune T cells lead to changes in DNA methylation patterns (5, 42, 43, 83, 84). The clearest example comes from SLE studies examining miRNAs. Several independent case-control studies found that *CD4+* T cells from SLE patients have overexpression of miR-21 and miR-148a, which correlates with significantly reduced DNA methylation levels in those cells (18, 42, 45).

Mechanistically, Pan et al. (2010) provided critical insights into the epigenetic dysregulation that characterizes systemic lupus erythematosus (SLE), focusing on the role of specific microRNAs (miRNAs) in altering DNA methylation patterns in lupus T cells (18). The study identified miR-21 and miR-148a as key contributors to the reduction in DNA methyltransferase 1 (DNMT1) expression, which in turn led to widespread DNA hypomethylation, a hallmark of aberrant gene expression in autoimmune diseases (18, 42, 45). MiR-148a was shown to directly bind to the 3’ coding region of DNMT1 mRNA, effectively repressing its translation (18). This direct interaction results in decreased synthesis of the DNMT1 protein, which is essential for maintaining DNA methylation patterns during cell division. Reduced DNMT1 levels impair the ability of T cells to sustain proper epigenetic silencing, leading to the derepression of genes normally kept in check by methylation (18, 42). In contrast, miR-21 affects DNMT1 expression indirectly. It targets RAS guanyl-releasing protein 1 (RASGRP1), a signaling molecule upstream of DNMT1 regulation. By downregulating RASGRP1, miR-21 disrupts a signaling cascade that ultimately leads to lower DNMT1 expression (18). Although indirect, this mechanism converges functionally with miR-148a to compound the reduction in DNMT1 protein levels. The downstream consequence of diminished DNMT1 activity is global DNA hypomethylation in CD4+ T cells from lupus patients (18, 42, 43). This epigenetic shift results in the aberrant overexpression of methylation-sensitive genes such as CD70 and LFA-1 (CD11a) (18, 45). Both genes are known to be involved in immune activation and T cell co-stimulation. CD70, a ligand for the TNF receptor family member CD27, plays a role in B cell activation and immunoglobulin production, while LFA-1 is involved in T cell adhesion and migration. Their overexpression promotes hyperactive immune responses, breaking self-tolerance and contributing directly to the autoimmune pathology observed in SLE (18, 45). The study also showed that treating lupus patient T cells *ex vivo* with inhibitors of miR-21 and miR-148a could *increase DNMT1 levels and partially reverse DNA hypomethylation*, bringing the methylation status of genes closer to that in healthy T cells (18).

This provides direct evidence that these miRNAs cause epigenetic alterations in T cells. Consistent findings were reported for miR-126 and miR-29b in lupus: these miRNAs are also elevated in SLE T cells and have been found to negatively regulate DNMT1 further reinforcing that multiple miRNA pathways converge on the DNA methylation machinery (42, 43, 45). In aggregate, SLE patients’ T cells appear to have a “perfect storm” of upregulated miRNAs (miR-21, 148a, 126, 29b, among others) that each contribute to undermining DNA methylation maintenance (18, 42, 43, 45). This hypomethylated state is a hallmark of lupus T cells and is known to lead to aberrant gene expression and autoreactivity (42, 45).

Other autoimmune diseases showed analogous patterns in T cells, though the specific miRNAs differed. For instance, in rheumatoid arthritis (RA), several studies noted altered T cell miRNA profiles with epigenetic consequences. One cross-sectional study found RA patients’ CD4+ T cells had lower expression of miR-146a (a miRNA with anti-inflammatory roles) compared to controls, and this was associated with increased expression of miR-146a targets involved in chromatin remodeling and inflammation (such as *IRAK1*, and *STAT1*; though DNA methylation was not directly measured in that study) (48, 49). The importance of miR-146a is highlighted by animal data: miR-146a-deficient mice develop a lupus-like autoimmune syndrome due to uncontrolled T cell activation (48).

Conversely, miR-155, a pro-inflammatory miRNA, tends to be overexpressed in autoimmune settings. In an included MS study, miR-155 was elevated in T cells from patients with active multiple sclerosis, which correlated with lower expression of the SOCS1 gene (a suppressor of cytokine signaling) and skewing toward a T_H17 phenotype (44, 46, 47). Animal experiments strongly support miR-155’s pathogenic role: miR-155 knockout mice are resistant to autoimmune encephalomyelitis, as their CD4+ T cells fail to efficiently differentiate into the pathogenic Th17 and Th1 subsets (44, 47).

O’Connell et al. showed that miR-155 directly promotes inflammatory T cell development and that its absence leads to an epigenetic state in T cells that is less permissive for IL-17 and IFN-γ gene expression (44, 46, 47). Thus, in both humans and mice, high miR-155 is associated with epigenetic changes (such as possibly increased permissive histone marks at cytokine loci, though not measured in all studies) that favor autoimmunity. Other miRNAs implicated across diseases include miR-31 (upregulated in psoriasis T cells, affecting FOXP3 methylation) (55), miR-24 and miR-210 (dysregulated in various contexts, with putative epigenetic targets) (54, 55). In summary, a broad finding is that autoimmune T cells exhibit miRNA expression changes that either drive DNA hypomethylation or other epigenetic alterations promoting pro-inflammatory gene expression, and experimental modulation of these miRNAs can often reverse those epigenetic changes.

Long non-coding RNAs in T cell epigenetic regulation: The included studies also shed light on how lncRNAs contribute to T cell epigenetic states in autoimmunity. One recurrent lncRNA is GAS5 (Growth Arrest Specific 5), which emerged in multiple SLE-focused studies. GAS5 is normally highly expressed in quiescent lymphocytes and acts as a decoy for the glucocorticoid receptor, among other functions. In SLE patients, GAS5 expression in CD4+ T cells was consistently found to be significantly decreased relative to healthy controls (27, 73).

Low GAS5 levels were correlated with higher T cell activation status and IL-2 production in lupus. Importantly, one case-control study demonstrated a functional link: overexpressing GAS5 in lupus patient T cells *in vitro* (using a plasmid or viral vector) inhibited T cell activation and even reduced the “self-reactivity” of those lupus T cells (measured by autoreactive T cell responses) (27).

Mechanistically, GAS5 was found to act through a ceRNA (competitive endogenous RNA) mechanism in T cells: it sponges miR-92a-3p, a microRNA that was elevated in SLE T cells, ßthereby preventing miR-92a from repressing its target E4BP4 (also known as NFIL3) (27, 52).

E4BP4 is a transcriptional repressor involved in restraining T cell activation. Thus, in lupus T cells, GAS5 downregulation leads to unchecked miR-92a activity, which in turn downregulates E4BP4, relieving repression on activation-induced genes. While the study did not directly measure epigenetic marks, E4BP4 can influence chromatin accessibility at certain gene promoters; the restoration of GAS5 likely re-established some repressive epigenetic control via E4BP4. From a broader perspective, GAS5’s effect illustrates how lncRNAs can modulate T cell epigenetics indirectly by targeting miRNAs and transcriptional repressors. Other lncRNAs found to be downregulated in lupus T cells included linc0597 and lnc-DC, both of which have been associated with immune cell regulation (27, 73, 78). While their functions in T cells are not fully elucidated, the consistent decrease of multiple lncRNAs suggests a pattern where pro-regulatory lncRNAs are lost in autoimmunity, contributing to a more permissive epigenetic environment for T cell over-activation (27, 73, 78, 80).

Some lncRNAs act as positive regulators of inflammatory T cell programs. For example, an interesting discovery from an animal study was lncRNA-GM, a novel intergenic lncRNA identified in a mouse model of autoimmune neuroinflammation (EAE). LncRNA-GM was found to be highly induced under T_H17-skewing conditions and was required for full T_H17 differentiation. Mechanistically, lncRNA-GM binds to the mRNA of *Foxo1* (a transcription factor that normally restrains T_H17 differentiation) and promotes its phosphorylation and inactivation. Foxo1 inactivation leads to enhanced IL-23 receptor expression and a feed-forward loop that stabilizes the T_H17 lineage. The lncRNA-GM study showed that knocking down lncRNA-GM in CD4+ T cells resulted in *increased* binding of Foxo1 to target genes (like *Il17* locus), causing more repressive chromatin at those loci and impairing T_H17 development. *In vivo*, mice lacking lncRNA-GM had attenuated autoimmune pathology (75).

This indicates that lncRNA-GM epigenetically skews T cells toward a pathogenic phenotype by interfering with a chromatin-modifying transcription factor (Foxo1). Similarly, the previously mentioned Flicr lncRNA in Tregs provides a contrasting example: Flicr *decreases* chromatin accessibility at the *Foxp3* gene, thereby limiting Treg differentiation.

In a NOD mouse model, higher Flicr levels were associated with reduced Foxp3 expression and accelerated autoimmune diabetes, whereas Flicr deficiency led to more robust Treg development and disease protection (74).

Together, these studies highlight a principle that lncRNAs can orchestrate the epigenetic state of T cell lineage-specification genes (like *FOXP3*, *RORγt*) and thus dictate T cell fates in autoimmune responses.

Another prominent lncRNA is MALAT1, studied in the context of multiple autoimmune diseases. MALAT1 was generally found to be *upregulated* in inflammatory T cells. A study showed higher MALAT1 in peripheral blood T cells during active RA and SLE flares compared to remission or healthy states) (27, 76, 77). MALAT1 is known to interact with epigenetic regulators (such as the Polycomb repressive complex component EZH2) and splicing factors (27, 76).

While specific MALAT1 mechanistic studies in T cells were limited, its consistent dysregulation and known functions suggest it likely impacts the expression of multiple immune genes via epigenetic modulation (for instance, MALAT1 can sequester EZH2 away from certain loci, affecting H3K27 methylation patterns) (27, 76). Supporting this, the review by Mohan et al. noted that MALAT1 influences epigenetic modifications and gene expression networks in autoimmunity (77).

Some included studies measured MALAT1 levels as a biomarker; for example, higher MALAT1 correlated with higher disease activity in SLE (and interestingly, MALAT1 levels dropped in patients who responded to immunosuppressive therapy, hinting at a connection between MALAT1 expression and the underlying epigenetic/inflammatory state) (27).

Epigenetic consequences beyond DNA methylation: While DNA methylation was a common focus, a few studies looked at histone modifications in T cells as influenced by ncRNAs. One animal study examined *miR-10a* in Tregs and found that miR-10a promotes Treg stability by targeting *Bcl-6* and *Sirt1*, leading to increased acetylation (active chromatin) at the *Foxp3* locus (53). Conversely, in a related human context, low miR-10a in lupus Tregs was associated with reduced FOXP3 acetylation and unstable Tregs (53). Another study (in MS) found that *miR-21* overexpression in CD4+ T cells was associated with loss of the repressive mark H3K27me3 at the *IL17A* gene, although that study inferred the effect rather than directly proving causation (44). On the lncRNA side, one included study reported that NEAT1, a pro-inflammatory lncRNA, can bind the RNA-binding protein NONO and alter the chromatin structure at cytokine gene promoters in T cells (this was shown *in vitro* with T cell lines) (50). In that study, silencing NEAT1 led to increased H3K27me3 at the *IFNG* promoter and lower IFN-γ production, suggesting NEAT1 helps maintain an open chromatin state at inflammatory gene loci (50). While such findings need further validation, they illustrate the diverse mechanisms by which ncRNAs regulate histone marks and chromatin architecture in T cells.

In summary, across the studies reviewed, miRNAs and lncRNAs consistently emerged as key regulators of T cell epigenetic status. MicroRNAs predominantly acted by fine-tuning the levels of epigenetic enzymes or key signaling proteins, thereby indirectly shaping DNA methylation and histone modification landscapes. LncRNAs acted through a variety of mechanisms—guiding chromatin modifiers, scaffolding transcriptional complexes, sequestering miRNAs, or modulating transcription factor activity—to exert either repressive or activating epigenetic effects on target genes. The net impact in autoimmune diseases is an imbalance where pro-inflammatory genes in T cells become epigenetically de-repressed (through DNA hypomethylation or open chromatin configuration), and regulatory genes (like *FOXP3* or *IL-2* in Tregs) may be inadequately expressed due to epigenetic silencing. NcRNA dysregulation is a unifying explanation for how these epigenetic abnormalities arise. Importantly, many studies demonstrated that correcting ncRNA levels can reverse epigenetic and functional defects in T cells (such as using miRNA inhibitors to restore methylation, or adding back a lncRNA to reinstate gene repression (14, 18, 19, 27, 44, 47, 48, 50, 52, 53, 66, 73, 75–78, 80). This not only solidifies the causal role of ncRNAs but also hints at therapeutic possibilities.

## Discussion

**Figure 9:**
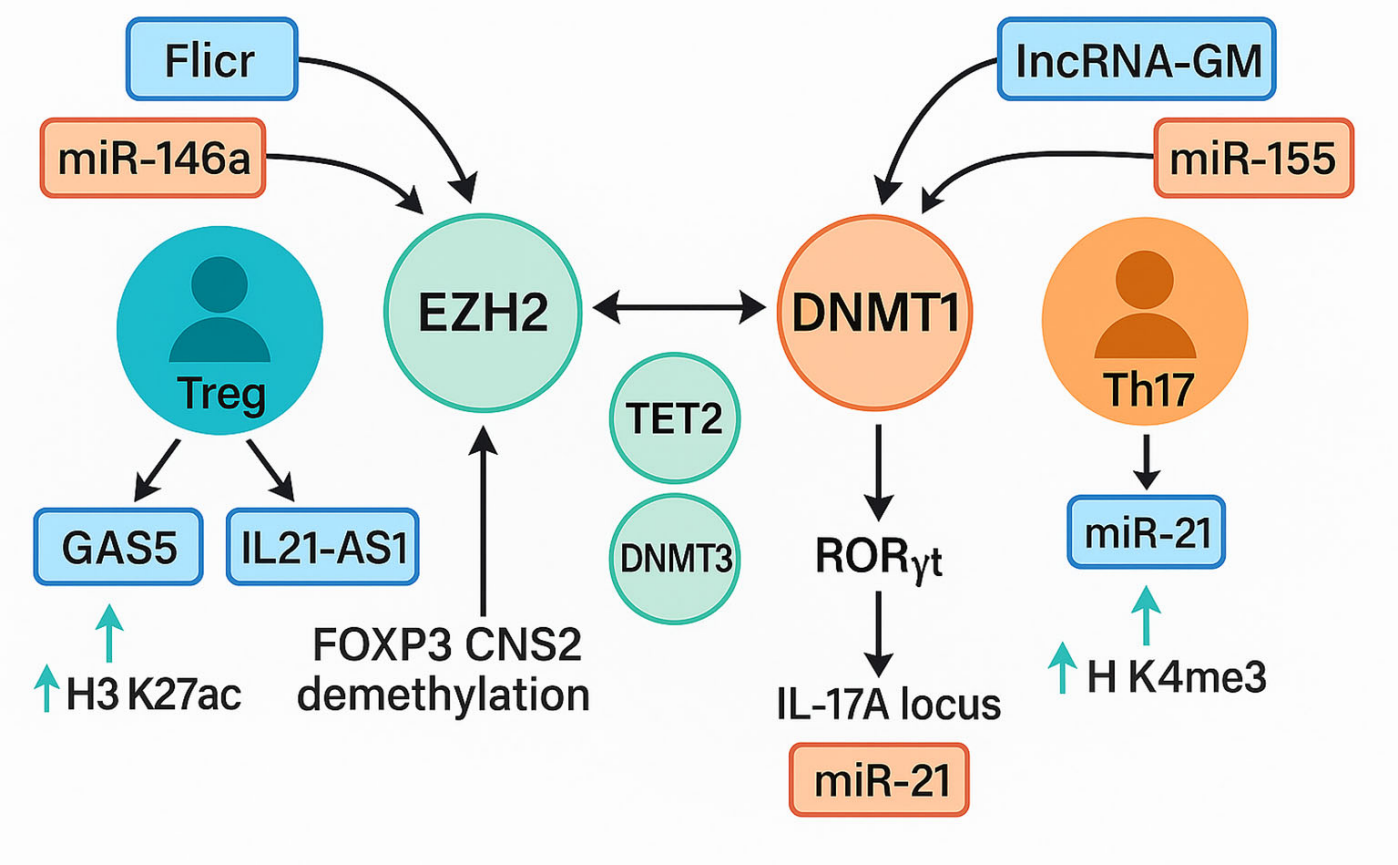
Interplay between ncRNA signaling and epigenetic modifiers in treg and Th17 differentiation. A schematic representation of ncRNA-mediated epigenetic regulation of T-cell fate. The Treg axis (blue–green) involves lnc-Flicr, GAS5, and miR-146a, which stabilize FOXP3 expression through EZH2, TET2, and DNMT3A, promoting H3K27ac enrichment and FOXP3 CNS2 demethylation that enhance regulatory T-cell stability. IL21-AS1 further contributes to maintaining immune tolerance by modulating chromatin accessibility at cytokine loci. The Th17 axis (orange–red) features lncRNA-GM and miR-155, which repress DNMT1 and activate RORγt, leading to IL17A transcription via H3K4me3 enrichment and chromatin relaxation at inflammatory loci. Cross-regulation among EZH2, DNMT1, and TET2 integrates both pathways, defining the epigenetic equilibrium that governs T-cell plasticity and autoimmune susceptibility Treg, regulatory T cell; Th17, T helper 17 cell; FOXP3, forkhead box P3; EZH2, enhancer of zeste homolog 2; TET2, ten-eleven translocation methylcytosine dioxygenase 2; DNMT1, DNA methyltransferase 1; DNMT3A, DNA methyltransferase 3 alpha; RORγt, retinoic acid receptor–related orphan receptor gamma t; IL17A, interleukin-17A; H3K27ac, histone 3 lysine 27 acetylation; H3K4me3, histone 3 lysine 4 trimethylation; lncRNA, long non-coding RNA; miRNA, microRNA; GAS5, growth arrest–specific 5; Flicr, FOXP3-associated long intergenic non-coding RNA; IL21-AS1, interleukin-21 antisense RNA 1; lncRNA-GM, long non-coding RNA-GM.

This systematic review integrated evidence from forty-six eligible investigations to clarify how non-coding RNAs remodel the epigenetic landscape of T cells in immune-mediated disease (2, 14, 27, 53). Taken together, the reviewed literature supports a unifying model in which ncRNAs converge on three principal epigenetic axes: (i) suppression or recruitment of DNA methyltransferases and demethylases, (ii) modulation of histone-modifying complexes such as PRC2 and CBP, and (iii) control of chromatin accessibility at lineage-defining loci such as FOXP3, IL21, and RORγt. Through these pathways, dysregulated ncRNAs destabilize the epigenetic checkpoints that normally enforce T cell tolerance, thereby fueling autoimmune pathology. These interactions are schematically shown in **Figure 9**, which delineates how dysregulated non-coding RNAs modulate epigenetic regulators to recalibrate the equilibrium between regulatory T cell (Treg) and Th17 lineage differentiation.

Across the included studies, the same overarching message emerged: microRNAs, long noncoding RNAs, and to a lesser extent circular RNAs operate as upstream regulators that can either destabilize or reinforce the heritable transcriptional program of T lymphocytes (14, 19, 66). Most of the thirty purely human studies documented characteristic ncRNA expression shifts in patient-derived T cells—most often an increase in microRNAs such as miR-21, miR-148a, miR-155, miR-125b, or miR-142-3p/5p (18, 45, 49, 67), a decrease in counter-regulatory microRNAs like miR-146a (48), and selective loss of tolerogenic lncRNAs such as IL21-AS1, GAS5, or LINC01882 (13, 73, 80). These molecular signatures almost invariably paralleled epigenetic abnormalities, for example hypomethylation of pro-inflammatory cytokine loci, reduced H3K27 trimethylation at FOXP3 or IL2 promoters, and heightened histone acetylation at enhancer elements driving IL-17 or IL-21 (11, 13, 42, 43). When the four quasi-experimental human papers manipulated ncRNA levels ex vivo, the direction of causality became clearer: silencing over-expressed microRNAs restored DNMT1 abundance and partially normalized DNA methylation, whereas re-expression of depleted lncRNAs re-established repressive histone marks and dampened autoreactive gene expression (18, 45, 48, 73).

The seven animal-only and nine combined human–murine studies provided a stringent mechanistic counterpoint. Genetic ablation of miR-155, miR-142-3p, or miR-210, for example, shielded mice from experimental autoimmune encephalomyelitis, lupus-like nephritis, or psoriasis-like skin inflammation (49, 51, 55), and these phenotypic improvements coincided with more repressive chromatin at signature Th1/Th17 loci (12, 72). Conversely, forced over-expression of circ_003912 (77) or sustained knock-down of Tet2 via miR-142-3p (51) skewed the Th17/Treg balance, produced DNA hypermethylation at the FOXP3 CNS2 enhancer, and precipitated aggressive autoimmunity (51, 77). Such concordance between patient correlations and *in-vivo* causality substantially strengthens the inference that ncRNA dysregulation is an initiating rather than a secondary event in immune pathology.

Mechanistically, a striking convergence on DNA-methylation homeostasis emerged. At least eight distinct up-regulated microRNAs in lupus, rheumatoid arthritis, or multiple sclerosis targeted DNMT1 directly or indirectly, collectively enforcing global or locus-specific hypomethylation of genes such as CD70, ITGAL, or IL13 (18, 42, 43, 45, 49, 62, 69, 74). Parallel lncRNA deficits relieved PRC2-mediated repression or diminished TET2 recruitment, further destabilizing the methylome (13, 51, 73). Equally compelling were examples of miRNA–lncRNA crosstalk: reduced GAS5 or IL21-AS1 removed sponges that normally sequester pro-inflammatory microRNAs, amplifying their impact on chromatin modifiers (13, 73, 80). Context specificity was another recurring theme. Flicr sustained FOXP3 accessibility uniquely in regulatory T cells (21), whereas lncRNA-GM (28) and miR-92a (52) orchestrated enhancer acetylation in Th17 and Tfh precursors respectively, reinforcing subset fidelity.

Beyond microRNAs, an expanding body of evidence implicates long non-coding RNAs (lncRNAs) and circular RNAs (circRNAs) as integral components of the epigenetic circuitry governing T cell differentiation and effector function. LncRNAs such as *IL21-AS1*, *GAS5*, *Flicr*, and *MALAT1* exert regulatory effects through distinct molecular modalities, including recruitment of chromatin-modifying complexes, modulation of enhancer accessibility, and sequestration of microRNAs within competing endogenous RNA networks. For example, *IL21-AS1* interacts with hnRNPU to recruit the coactivator CBP to the *IL21* promoter, thereby promoting histone acetylation and transcriptional activation of cytokine genes, whereas *Flicr* and *GAS5* attenuate T cell activation and maintain regulatory T cell stability by restricting chromatin accessibility at the *FOXP3* locus and limiting transcriptional noise. These lncRNA-mediated effects exhibit pronounced cell type and activation specificity, indicating that they operate within localized epigenetic microenvironments rather than through globally conserved pathways.

Emerging evidence also highlights the contribution of circular RNAs, such as *circ_003912* and *circ_0001320*, which can function as molecular sponges for multiple miRNAs, thereby stabilizing the expression of target transcripts and indirectly influencing chromatin remodeling enzymes. Nevertheless, current circRNA studies remain largely correlative and rely predominantly on bulk transcriptomic analyses without direct interrogation of chromatin topology. Future investigations integrating CRISPR-based perturbation with single-cell chromatin accessibility assays (such as ATAC-seq and CUT&Tag) will be essential to establish causality and delineate the hierarchical relationships among miRNAs, lncRNAs, and circRNAs. Collectively, these findings support a model in which non-coding RNAs form a multilayered regulatory network that modulates the epigenetic landscape of T cells in a context-dependent manner, but the mechanistic hierarchy and temporal dynamics of these interactions remain incompletely understood. The integration of lncRNAs and circRNAs into this regulatory paradigm suggests that ncRNA-mediated epigenetic control extends beyond post-transcriptional repression to encompass chromatin-level remodeling, thereby offering new mechanistic entry points for therapeutic intervention.

These mechanistic insights translate readily into clinical possibilities. Circulating or cell-associated ncRNA signatures, particularly elevated miR-155, miR-21, and diminished GAS5 or miR-146a, have already shown promise as biomarkers that track disease activity across lupus, RA, and MS cohorts (18, 48, 49, 73). More provocatively, several ex vivo and *in-vivo* studies demonstrated that antisense inhibition of pathogenic microRNAs or mimetic replacement of protective ncRNAs can recalibrate T-cell chromatin and temper inflammatory outputs, pointing toward oligonucleotide-based therapeutics (18, 45, 48, 49, 73). Because many ncRNAs display cell-type-restricted expression, such strategies might offer a precision lacking in global DNMT or HDAC inhibitors (12, 14). The feasibility of lipid-nanoparticle or exosome delivery into lymphoid tissue further supports the translational trajectory (27, 80). Therapeutic opportunities arising from this body of work extend beyond diagnostic biomarkers. The cell type–specific expression of ncRNAs offers a pathway to precision immunotherapy, where pathogenic miRNAs could be inhibited using antisense oligonucleotides, while tolerogenic lncRNAs could be restored with synthetic mimetics. Importantly, recent advances in lipid nanoparticle and exosome delivery systems raise the possibility of targeted administration directly into lymphoid compartments, potentially achieving T cell–restricted modulation while minimizing systemic immunosuppression. Early preclinical studies already demonstrate feasibility, positioning ncRNA-directed interventions as a promising new class of epigenetic therapies for autoimmunity.

## Limitations

Several methodological considerations should be taken into account when interpreting the findings of this systematic review. Many human studies included relatively small sample sizes, often fewer than fifty participants, which may limit the statistical power to detect subtle but biologically relevant associations between non-coding RNA expression patterns and epigenetic modifications in T cells. Small cohorts also increase susceptibility to type I and type II errors and may lead to overestimation or underestimation of effect sizes.

The predominance of cross-sectional study designs restricts the ability to establish temporal precedence between non-coding RNA dysregulation and disease onset or progression. Without longitudinal data, it is challenging to determine whether observed molecular changes are causative drivers of immune dysregulation or secondary consequences of chronic inflammation. Furthermore, many studies did not consistently control for potential confounding factors such as treatment status, age, sex, ethnicity, and differences in T cell subset composition. These variables can significantly influence both non-coding RNA expression and epigenetic states, potentially obscuring true mechanistic relationships.

Technical heterogeneity across studies further complicates synthesis and comparison of results. Variations in RNA isolation protocols, sequencing depth, normalization strategies, and epigenetic profiling techniques can introduce measurement bias and limit reproducibility. The lack of standardized bioinformatic pipelines for integrating transcriptomic and epigenomic datasets hampers the ability to generate comparable effect estimates and mechanistic insights across independent cohorts.

In animal studies, knockout and transgenic approaches, although powerful for establishing causality, may not fully recapitulate the physiological range of non-coding RNA modulation achievable in human disease. The absence of dose response evaluations in many experiments reduces the translational applicability of these findings. Additionally, reporting of randomization and blinding procedures was inconsistent, raising the possibility of outcome assessment bias.

Finally, the existing literature remains disproportionately focused on a limited set of well-studied microRNAs and long non-coding RNAs. This focus may obscure the contributions of less abundant or tissue specific non-coding RNAs with potentially significant epigenetic regulatory functions. The incomplete characterization of these molecules represents an important gap in the current understanding of T cell epigenetic regulation in autoimmune disease.

### Methodological variability and the need for standardization

The interpretation of current findings must account for substantial methodological heterogeneity across the included studies. Variability in RNA isolation methods, sequencing platforms, data normalization strategies, and analytical pipelines can introduce significant technical bias and limit comparability between datasets. Similarly, diverse approaches were employed to quantify epigenetic marks, including bisulfite sequencing, methylation arrays, chromatin immunoprecipitation, and ATAC sequencing, each with distinct sensitivity and genomic coverage. Such inconsistencies hinder the reproducibility of results and obscure subtle yet biologically meaningful effects.

Adoption of standardized protocols for sample processing, RNA and chromatin handling, sequencing library preparation, and bioinformatic integration is therefore essential to ensure methodological rigor and comparability across studies. Harmonization of these procedures will facilitate quantitative meta-analysis and enhance the interpretability of ncRNA-epigenome interactions in autoimmune diseases. Future investigations should also report detailed methodological metadata, including quality control metrics and normalization parameters, to promote transparency and reproducibility in this rapidly evolving field.

## Conclusions and future directions

This review integrates mechanistic and translational analyses to demonstrate that non-coding RNAs function as central architects of T cell epigenetic programming in autoimmune disease. These molecules orchestrate the regulation of DNA methylation, histone modifications, and chromatin accessibility in a coordinated manner, thereby stabilizing transcriptional identities that govern the balance between immune tolerance and pathogenic activation. The integration of these molecular processes forms a coherent regulatory architecture that links specific non-coding RNA perturbations to functional immune outcomes and clinical manifestations.

The accumulated body of research supports a conceptual shift in therapeutic strategy. Rather than broadly targeting epigenetic regulators, which can disrupt multiple cellular processes and cause substantial toxicity, future interventions could selectively modulate disease relevant non-coding RNAs to restore physiological epigenetic patterns. Such an approach combines molecular specificity with the potential for cell type selectivity, thereby enhancing therapeutic precision while minimizing off target effects. Achieving this goal will require a deeper understanding of the regulatory hierarchies in which non-coding RNAs operate, including their interactions with chromatin modifying enzymes, transcription factors, and structural components of the nucleus.

Future investigations should prioritize longitudinal designs that follow individuals from early or pre-clinical stages of autoimmunity through established disease and remission. This will enable researchers to distinguish early causal perturbations in non-coding RNA expression from secondary changes driven by chronic inflammation or treatment exposure. The use of single cell multi omics technologies will be essential to map cell type specific and temporal patterns of RNA chromatin interactions, providing unprecedented resolution of the spatial and functional organization of epigenetic regulation in immune cells. Functional genomic approaches such as CRISPR based activation and repression screens should be systematically employed to identify uncharacterized non-coding RNAs with previously unrecognized regulatory capacity in T cell biology.

Translational efforts should proceed in parallel with basic research to accelerate the movement of mechanistic discoveries into therapeutic applications. Preclinical testing of antisense oligonucleotides, small interfering RNAs, and synthetic RNA mimetics targeting validated disease associated non-coding RNAs will be essential to evaluate efficacy, specificity, and safety *in vivo*. Equally critical will be the refinement of delivery platforms, including lipid nanoparticles and engineered extracellular vesicles, to ensure targeted delivery to pathogenic immune subsets while avoiding systemic immune suppression. Integration of molecular diagnostics with therapeutic strategies could ultimately enable personalized treatment regimens guided by individual non- coding RNA and epigenetic profiles.

The convergence of high-resolution mechanistic mapping, innovative delivery technologies, and precision molecular targeting provides a realistic pathway toward restoring immune tolerance in autoimmune disease. Continued interdisciplinary collaboration between molecular biologists, immunologists, bioengineers, and clinical researchers will be essential to translate these insights into safe and effective therapies capable of modifying the disease course rather than merely controlling symptoms.

## Data Availability

The original contributions presented in the study are included in the article/**Supplementary Material**. Further inquiries can be directed to the corresponding authors.
