# Supplementary material for: The role of microRNAs and long non-coding RNAs in epigenetic regulation of T cells: implications for autoimmunity
Source: Front Immunol. 2025 Nov 25;16:1695894. doi: 10.3389/fimmu.2025.1695894 (PMC12685939; doi:10.3389/fimmu.2025.1695894)
Supplement: Supplementary file 1 [file DataSheet1.pdf]

Supplementary Table 1: A summary of included studies.

Reference	Study	Disease Studied	Population	Comparison	Outcomes
Wang-Renault et al. (50), 2018	Cross-sectional study	Primary Sjögren syndrome	44 female patients aged 40-78	17 healthy age- and ethnicity-matched controls	• 21 miRNAs significantly dysregulated in pSS CD4+ T cells (9 upregulated, 12 downregulated)<br>• 24 miRNAs significantly dysregulated in pSS CD19+ B cells (11 upregul[illegible]<br>• B-cell activating factor mRNA inversely correlated with expression of miR-30b-5p in pSS B cells
Hao et al. (78), 2021	Cross-sectional study	SLE<br>Rheumatoid arthritis (RA)	53 SLE patients (92.5% female) and 52 rheumatoid arthritis patients (67.3% female) aged 23-80	23 healthy controls	• IL21-AS1 expression in CD4+ T and B cells is decreased in SLE patients<br>• IL21-AS1 is positively correlated with IL-2 expression and the proportion of activated T follicular cells<br>• A allele of SNP rs62324212, located in the enhancer region of IL21-AS1, is associated with reduced IL21-AS1 expression<br>• Lower IL21-AS1 expression is negatively correlated with disease activity (SLEDAI score) and anti-dsDNA antibody positivity

Coit P et al. (84), 2022	Cross-sectional study	SLE	74 female patients	74 healthy age-, race-, and sex-matched CD4+ T cells	• Hypomethylation of the miR-17-92 promoter was observed in lupus patients, with miR-19b1 and miR-18a levels positively correlating with disease activity<br>• miR-18a target gene TNFAIP3, a negative regulator of NF-κB, was downregulated in lupus CD4+ T cells<br>• Although methylation quantitative trait loci (meQTL) overlapped with known lupus genetic risk loci (such as IRF7, and CFB), <1% of differentially methylated CpG sites were linked to meQTLs
Ruhrman et al. (63). 2018	Cross-sectional study	Multiple sclerosis (MS)	12 RR-MS patients (75% female) and 8 SP-MS patients (50% female) aged 26-63	12 healthy age- and sex-matched controls	• RR-MS patients showed hypermethylation at the VMP1/MIR21 locus in $CD4^{+}$ T cells, which correlated negatively with age and miR-21 expression, but not with MS risk genotype or smoking status<br>• Hypermethylation of MIR21 was associated with reduced miR-21 expression in RR-MS patients<br>• Reduced miR-21 levels in RR-MS were linked to upregulation of validated miR-21 target genes, including those involved in apoptosis and cell proliferation
Sebastiani et al. (60), 2017	Cross-sectional study	T1DM	13 adult patients	11 healthy controls	• miR-125a-5p was selectively upregulated in Treg cells from pancreatic lymph nodes (PLN) of T1D patients, but not in Treg or Tconv cells from peripheral blood or controls<br>• miR-125a-5p directly targeted TNFR2 and CCR2; overexpression correlated with reduced CCR2 levels in PLN Treg cells

					• CCL2, the ligand for CCR2, was highly expressed in pancreatic β-cells
Zhao M et al. (45), 2014	Cross-sectional study	SLE	42 patients aged 22-38	42 healthy CD4+ T cells	• Global hypomethylation in SLE $CD4^+$ T cells was linked to disease phenotype, affecting genes like NLRP2, CD300LB, and S1PR3<br>• miRNA and mRNA expression were inversely correlated with methylation, with dysregulated miRNAs (such as miR-126↑, miR-142-3p↓) targeting immune pathways<br>• 36 upregulated miRNAs were hypomethylated near CpG sites
Shu et al. (57), 2021	Cross-sectional study	Vogt-Koyanagi-Harada disease	10 adult patients	9 healthy CD4+ T cells	• Whole-transcriptome analysis identified 451 circRNAs, 433 miRNAs, and 5088 mRNAs differentially expressed in VKH $CD4^+$ T cells<br>• A circRNA–miRNA–mRNA (ceRNA) network was constructed, identifying immune-related genes (such as LAT, ZAP70, and ICOS) and regulatory circRNAs potentially modulating VKH pathogenesis<br>• hsa_circ_0001320 and hsa_circ_0001924 were validated by RT-qPCR as potential VKH biomarkers
Coit P et al. (85), 2016	Cross-sectional study	SLE	74 female patients aged 18-66	None mentioned	• Naive $CD4^+$ T cells in SLE exhibit epigenetic remodeling correlated with disease activity, showing hypomethylation of proinflammatory genes (such as IL4, IL5, IL13, RORγt, and BCL6) and hypermethylation of inhibitory genes (such as TGF-β, GATA3, and T-bet)<br>• EZH2 emerged as a key epigenetic regulator, with decreased miR-26a correlating with higher EZH2 activity during flares

					• DNA methylation changes in flares occurred prior to transcription
You et al. (47), 2021	Cross-sectional study	SLE	7 female patients	7 healthy age-matched controls	• lncRNA AC007278.2 was upregulated in SLE and shown to regulate immune and inflammatory genes CCR7, AZU1, and TNIP3<br>• AC007278.2 repressed CCR7 transcription by inhibiting its promoter activity; CCR7 and AC007278.2 expression were inversely correlated in SLE patients<br>• AC007278.2 modulates innate immunity and cytokine responses, suggesting a trans-acting mechanism
Liu D et al. (71), 2016	Cross-sectional study	SLE	20 adult patients	20 healthy CD3+ T cells	• miR-410 is downregulated in SLE $CD3^+$ and $CD4^+$ T cells, and its expression inversely correlates with IL-10 levels<br>• Overexpression of miR-410 suppresses IL-10 by directly targeting STAT3, reducing STAT3 and IL-10 expression in SLE T cells<br>• Silencing STAT3 mimics miR-410 effects, confirming STAT3 as a mediator of IL-10 regulation in $CD3^+$ T cells
Zhu Y et al. (43), 2019	Cross-sectional study	Primary immune thrombocytopenia	21 patients (76% female) aged 22-46	18 healthy controls	• 37 miRNAs were differentially expressed in Tregs from ITP patients, with 26 upregulated and 11 downregulated<br>• miR-155–5p, miR-146b–5p, and miR-142–3p were significantly downregulated in ITP Tregs, confirmed by qPCR<br>• Dysregulated miRNAs may impair Treg function, contributing to ITP pathogenesis

Ramanujan et al. (65), 2021	Cross-sectional study	SLE	21 adult patients	12 healthy age-, race-, and sex-matched controls	• Estrogen upregulates hsa-miR-10b-5p in T cells, which is higher in healthy women and SLE patients<br>• hsa-miR-10b-5p downregulates SRSF1 posttranscriptionally by targeting its 3′-UTR, reducing protein expression<br>• Elevated hsa-miR-10b-5p in SLE may contribute to T cell dysfunction by suppressing SRSF1, linking estrogen to autoimmunity
Chen et al. (83), 2017	Cross-sectional study	Ankylosing spondylitis (AS)	15 adult patients	10 healthy controls	• miR-10b is increased in Th17 cells cultured from patients with AS<br>• miR-10b inhibits Th17 responses and is transiently induced during Th17 differentiation<br>• miR-10b inhibits MAP3K7 through 30UTR binding; MAP3K7 silencing in Th17 cells inhibits IL-17A production
Zhao M et al. (46), 2018	Cross-sectional study	SLE	36 patients aged 21-34	36 healthy CD4+ T cells	• BDH2 is downregulated in SLE CD4+ T cells, leading to increased intracellular iron, elevated DNA hydroxymethylation, and reduced DNA methylation<br>• miR-21 directly targets and suppresses BDH2, promoting epigenetic dysregulation and overexpression of autoimmune-related genes (CD70, CD11a, CD40L, perforin)<br>• Restoring BDH2 or inhibiting miR-21 reverses iron accumulation and DNA hypomethylation

Schiavinato et al. (61), 2017	Cross-sectional study	T1DM	10 new-onset T1DM children	10 healthy age-matched children	• TGF-β and atRA enhance $FOXP3^{+}$ iTreg generation from naïve umbilical cord T cells more efficiently than IL-2 alone<br>• 32 microRNAs induced by TGF-β/atRA post-transcriptionally downregulate IL-6/JAK/STAT and mTOR pathways<br>• miR-1299 and miR-30a-5p directly downregulate IL6R/IL6ST and increase $FOXP3^{+}$ cells
Acevedo et al. (86), 2020	Cross-sectional study	Atopic dermatitis	10 male patients aged 20-49	10 healthy CD4+ T cells	• IL13 hypomethylation drives increased expression in $CD4^{+}CLA^{+}$ T cells from AD patients<br>• Sixteen miRNAs are differentially expressed in AD, targeting immune signaling pathways including MAPK, TGF-β, and protein ubiquitination<br>• Integrated network analysis reveals distinct miRNA-CpG communities that differentiate AD from healthy controls
Xia et al. (48), 2018	Cross-sectional study	Rheumatoid arthritis	20 patients (90% female) aged 26-77	20 healthy CD3+ T cells	• MiR-128-3p is upregulated in RA T cells and correlates with increased inflammation (IL-6, IL-17) and reduced TNFAIP3 expression<br>• Knockdown of MiR-128-3p suppresses T cell activation and NF-κB signaling by upregulating TNFAIP3, reducing CD69/CD25 expression and pro-inflammatory cytokines<br>• In vivo, MiR-128-3p inhibition alleviates arthritis severity

Huang et al. (74), 2021	Cross-sectional study	Erosive oral lichen planus	65 patients (51% male) aged 42-49	70 healthy CD4+ T cells	• circ_003912 is upregulated in ELOP CD4$^+$ T cells, sponging miR-1231, miR-31, and miR-647 to increase FOXP3 and miR-146a while suppressing TRAF6<br>• circ_003912 modulates inflammatory cytokines, decreasing IL-4 and IL-10 while increasing IFN-γ and IL-2, and alters Treg counts and cell proliferation<br>• FOXP3 is directly targeted by miR-1231, miR-31, and miR-647
Heyn et al. (77), 2016	Cross-sectional study	Neuropathic pain	11 patients (64% female) aged 42-66	9 healthy CD4+ T cells	• miR-124a and miR-155 are elevated in neuropathic pain and directly suppress SIRT1 in CD4$^+$ T cells<br>• SIRT1 knockdown or miR-124a/miR-155 overexpression enhances Foxp3 expression, promoting Treg differentiation<br>• Treg levels are elevated in neuropathic pain, suggesting that miRNA-mediated downregulation of SIRT1 drives an anti-inflammatory T cell response
Elkhodiry et al. (81), 2023	Cross-sectional study	Multiple sclerosis	25 RRMS (Mean Age 39, 8:17 M:F Ratio)	10 Controls (Mean Age 30, 3:7 M:R Ratio), Healthy CD8+ T-Cells	• Knockdown of ICAM1/ ITGB2 via siRNA resulted in decreased miR-155 expression<br>• miR-155, ICAM1, and ITGB2 are potential therapeutic targets/biomarkers for RRMS<br>• miR-155 Overexpression in CD8+ T cells downregulated ICAM1, ITGB2, perforin, and granzyme B in all RRMS treatment subgroups

Liu L et al. (69), 2016	Cross-sectional study	Immune thrombocytopenia	46 ITP Patients (19 M, 27 F), Median Age 30	39 Healthy Controls (17M, 22F), Median Age 27	• ITP patients showed lower expression of miR-146a, miR-326, and miR-142-3p compared to healthy controls<br>• miR-142-3p and miR-146a expression levels were negatively correlated with increased Th17 cell frequency<br>• miR-146a levels were positively correlated with Treg frequency and platelet count
Kanduri et al. (72), 2015	Cross-sectional study	Autoimmune disease/allergy	Healthy neonates born in Turku University Central Hospital	Healthy CD4+ T Cells (Naive/Thp, Th0)	• Discovered 136 Th1-specific and 181 Th2-specific lncRNAs, with a positive correlation between expression of lineage-specific lncRNAs and nearby genes<br>• Lineage-specific genes and lncRNAs are enriched for enhancer (H3K4me1) and promoter (H3K4me3 and H3K27ac) marks<br>• SNPs associated with disease were enriched near Th1/Th2-specific genes and lncRNAs.
Syrett et al. (56), 2019	Cross-sectional study Experimental animal study	SLE	13 pediatric SLE patients, 2-5 mice per experiment	10 healthy age-matched controls; Healthy CD4+ and CD8+ T cells; WT mice	• Naïve lymphocytes lose heterochromatin markers such as H3K27me3 on Xi, but they reappear after activation<br>• Reactivation of X-linked gene expression in SLE patient $CD4^+/CD8^+$ T cells was accompanied by altered expression of chromatin modifiers like YY1 and hnRNPU<br>• Epigenetic instability of Xi in T cells may increase X-linked gene dosage, providing a molecular explanation for the female predominance in SLE

Pan et al. (18), 2010	Cross-sectional study Experimental animal study	SLE	36 SLE Patients	30 Control Patients; Healthy CD4$^+$ T cells, Untreated cells	• SLE patients and MRL/lpr lupus-prone mice had significant overexpression of miR-21/miR-148a in CD4$^+$ T cells relative to controls<br>• miR-21 indirectly downregulates DNMT1 by targeting upstream regulator RASGRP1<br>• miR-148a directly binds DNMT1 to suppress its translation, leading to reduced DNMT1 protein levels and global DNA hypomethylation
Liu L et al. (70), 2022	Cross-sectional study Experimental animal study	SLE	SLE Patients: 63	Healthy Controls: 33, Healthy CD4+ T cells, WT mice Wild-type mice compared to knock-in mice, Untreated/negative control CD4+ T cells under same induction conditions	• In CD4$^+$ T cells from SLE patients, lncRNA IL21-AS1 was significantly upregulated and shown to enhance IL-21, IFN-γ, and IL-17 expression<br>• IL21-AS1 binds hnRNPU, which recruits the coactivator CBP to the IL21 promoter, leading to increased H3 acetylation and transcriptional activation of IL-21<br>• Blocking IL21-AS1/hnRNPU reduced IL-21 expression and Tfh frequency, suggesting IL21-AS1 as a potential target for personalized SLE therapies/biomarkers
Zhu H et al. (44), 2024	Cross-sectional study Experimental animal study	Immune thrombocytopenia	7 ITP patients, 12 Mice	5 Healthy Controls, 6 Mice; Healthy CD4+ T cells, Untreated cells	• CD4$^+$ T cells from ITP patients upregulated miR-641, directly suppressing STIM1/SATB1 and disrupting Th17/Treg balance<br>• Overexpression of miR-641 increased IL-17A production and reduced FOXP3$^+$ Treg cells<br>• miR-641 rebalanced Th17/Treg proportions and suppressed IL-17A/IFN-γ secretion

Wang J et al. (53), 2016	Case control study	SLE	Immortalised B-cell lines: SLE n = 5 pediatric females;	Immortalised B-cell lines: Healthy Female Controls n = 3; Healthy $CD4^+/CD8^+$ T cells and B cells; WT Mouse Lymphocytes, Untreated cells	• Positive correlation between HDAC4/7 levels and Th17-associated cytokines<br>• HDAC4 uses transcription factor JunB to promote expression of Th17 signature genes<br>• HDAC7 via Aiolos/SMRT/NCoR-HDAC3 complex represses negative regulators like Il2<br>• Hdac4-/Hdac7-deficient $CD4^+$ T cells and treatment with TMP269 suppressed Th17-mediated colitis in mouse models, decreasing IL-17A/F and other pathogenic cytokines
Conteduca et al. (87), 2021	Case control study	Alopecia areata	184 AA patients	200 Controls; Healthy PBMCs	• The genotype carrying the rs4404254 3'UTR alleles and reduced inducible costimulator molecules (ICOS) were more commonly seen in AA patients<br>• miR-1276 significantly suppressed ICOS expression by binding to the 3′UTR of ICOS mRNA<br>• miR-101 and miR-27b were upregulated and miR-103 and miR-2355-3p were downregulated in PBMCs
Kalla et al. (73), 2020	Case control study	IBD	Stage 1 Cohort (24 IBD Patients(9 CD, 14 UC, 1 IBDU); Stage	Stage 1 Cohort (8 Healthy Controls, Healthy CD4+ T cells); Stage 2 Cohort(98 Healthy Patient Controls)	• miR-1307-3p was upregulated in IBD, especially UC, but was not associated with CD location or behavior<br>• miR-1307-3p, miR-3615, and miR-4792 predicted treatment escalation, with strongest performance in Crohn's disease

			2 Cohort (98 CD, 97 UC, 1 IBDU)		• Combined miRNA, age, and albumin models improved prognosis, identifying high-risk CD patients (83% escalation at 1 year)
Hosokawa et al. (76), 2015	Case control study	Acquired aplastic anemia<br>Myelodysplastic syndrome<br>Sickle cell disease	AA Patients: 15, MDS: 5, SCD: 5	11 Healthy Controls; Healthy CD4/CD8+ cells	• miR-126-3p and miR-223-3p were downregulated in CD4+ T effector memory cells<br>• miR-126-3p, miR-145-5p, and miR-223-3p were downregulated in CD8+ T effector memory and terminal effector cells<br>• MYC and PIK3R2—targets of miR-145-5p and miR-126-3p, respectively—were upregulated in CD4+ and CD8+ T cells
Garchow et al. (80), 2021	Cross-sectional study<br>Experimental animal study	SLE	6 Lupus Patients	4 Healthy Controls; Healthy CD4+ T cells	• miR-210 and HIF-1α are overexpressed in CD4+ T cells from lupus-prone mice and lupus patients, with miR-210 correlating strongly with disease activity<br>• Expression is lineage-specific, with CD8+ and CD19+ cells showing minimal change in miR-210 and HIF-1α<br>• RORγt is significantly upregulated in lupus CD4+ T cells and correlates with HIF-1α expression
Serr et al. (59), 2016	Cross-sectional study	T1DM	17 Children with Recent Onset of Islet Autominunty,	23 Healthy Controls; Healthy CD4+ T-Cells, Untreated Cells	• miR-92a is upregulated in early islet autoimmunity and promotes TFH precursor expansion by suppressing PTEN–Foxo1–KLF2 signaling<br>• miR-92a mimics boost TFH cells and Th2 cytokines, while antagomirs inhibit TFH induction and enhance Tregs in vitro

	Experimental animal study		19 with Perisistent Islet Autoimmunity, 17 with Long-term Islet Autoimmunity		• In vivo miR-92a blockade reduces TFH cells and pancreatic infiltration, preserving β-cell integrity in NOD mice
Liu R et al. (68), 2017	Case control study Experimental animal study	Multiple sclerosis	37 MS (RRMS) Patients	40 Healthy Controls; Healthy CD4+ T Cells	• miR-15b expression is downregulated in MS patients and in mice with experimental autoimmune encephalomyelitis<br>• miR-15b suppresses Th17 differentiation in vitro and in vivo, reducing IL-17–driven autoimmunity<br>• OGT is a direct target of miR-15b, linking it to modulation of RORγt via NF-κB glycosylation
Scherm et al. (62), 2019	Quasi-experimental study Experimental animal study	T1DM	20 Mice for IAA- and IAA+, 5 Diabetic; 8 Humans with recent onset or long-term T1D	22 Human Controls; Healthy Naive CD4+ T cells, Untreated cells	• miR142-3p is highly enriched in RISC of human CD4+ T cells and induced in islet autoimmunity<br>• Inhibition of miR142-3p enhances Treg induction and stability by increasing Tet2 expression and promoting Foxp3 CNS2 demethylation<br>• Systemic miR142-3p inhibition in non-obese diabetic mice with islet autoimmunity reduces pancreatic T cell infiltration, improves Treg stability, and lowers autoimmunity markers

Peng et al. (66), 2020	Analytical cross-sectional study	Rheumatoid arthritis	RA: 31 (8M/23F), OA: 8 (2M/6F)	Healthy: 30 (6M/24F); Healthy CD4+ T cells, Untreated cells	• IFNG-AS1 is upregulated in RA patients and correlates with disease severity markers (RF, ESR, CRP) and IFNG and T-bet expression levels<br>• T-bet directly regulates IFNG-AS1 transcription in $CD4^{+}$ T cells, shown by siRNA knockdown experiments<br>• IFNG-AS1 shows diagnostic potential with an AUC of 0.815, high specificity (96.8%), and moderate sensitivity for RA
Torri et al. (54), 2017	Quasi-experimental study	Psoriasis	Psoriasis patients (n=39)	Healthy CD4+ T cells from donors (n=38)	• EV-associated miRNAs distinguish Th1, Th17, and Treg cells, with miR-146a-5p, miR-150-5p, and miR-21-5p enriched in Treg-derived EVs<br>• Treg-derived EVs suppress CD4+ T cell activation by downregulating IRAK2, STAT1, and c-Myb and reducing proliferation<br>• miR-106a-5p and miR-150-5 are elevated in psoriasis sera, some of which normalize with anti-TNF therapy
Lu et al. (67), 2020	Analytical cross-sectional study	Acquired aplastic anemia	3 AA patients	3 healthy controls	• 25 dysregulated miRNAs and 199 negatively correlated mRNAs were identified in bone marrow T cells of AA patients, with hsa-mir-34a-5p, hsa-mir-195-5p,and hsa-mir-424-5p targeting the most mRNAs<br>• Functional and pathway enrichment analyses revealed the role of MAPK signaling, chromatin regulation, and myeloid differentiation in cancer<br>• miR-34a-5p, miR-195-5p, and miR-424-5p target hub genes like VEGFA and CCND1

Gong et al. (79), 2021	Quasi-experimental study	Primary Sjögren syndrome	13 pSS patients	13 healthy controls (CD4+ T cells and untreated pSS cells)	• Increased miRNA-7150 and miRNA-5096 and decreased miRNA-125b-5p and miRNA-22-3p levels in pSS CD4+ T cells were reversed by MSC treatment<br>• Proliferation of CD4+ T cells and CD4+ IFN-γ+ cells and miRNA-125b-5p and miRNA-155 expression, and supernatant IFN-γ secretion were associated with disease
Houtman et al. (75), 2018	Quasi-experimental study	Rheumatoid arthritis	354 RA patients (methylation); 137 RA patients (expression)	335 Healthy Controls (methylation), 59 Healthy Controls (expression);	• SNPs in the PTPN2 locus are highly correlated with DNA methylation at four downstream CpG sites and lncRNA LINC01882 expression downstream of these CpG sites<br>• anti-CD3/CD28 activated naïve CD4þ T cells downregulate the expression of LINC01882<br>• LINC01882 knockdown upregulates transcription factor ZEB1 and kinase MAP2K4, both involved in IL-2 regulation
Tang et al. (55), 2015	Quasi-experimental study	SLE	7 SLE patients (F, age 16–35)	6 healthy controls; Untreated Lupus CD4+ T cells, Healthy CD4+ T cells	• 101 upregulated microRNAs and 77 downregulated microRNAs were identified in MPA-treated lupus CD4þT cells, of which miR-142-3p/5p and miR-146a expression was significantly increased<br>• MPA-treated CD4+ T cells showed enriched H4 acetylation in the putative miRNA-142 regulatory region and H3 acetylation in the putative miRNA-146a regulatory region

Ding et al. (82), 2012	Quasi-experimental study	SLE	30 SLE Patients	20 Healthy Controls; Healthy CD4+ T cells	• miR-142-3p and miR- 142-5p are significantly downregulated in SLE CD4+ T cells<br>• miR-142-3p/5p levels directly inhibit SLE-related targets signaling lymphocytic activation molecule–associated protein (SAP), CD84, and interleukin-10 (IL-10)<br>• Decrease in miR-142 expression correlated with changes to histone modifications and DNA methylation levels upstream of the miR-142 precursor sequence
Severin et al. (58), 2016	Quasi-experimental study	Multiple sclerosis	22 MS Patients	16 Healthy Controls; Healthy PBMCs, Untreated cells	• Multiple sclerosis (MS) patients exhibit decreased TGFβ signaling in naïve $CD4^+$ T cells<br>• miRNAs (such as miR-27b, miR-103a, miR-128, and miR-141, let-7b) directly bind to TGFBR1 and SMAD4 3'UTRs, reducing their protein expression in vitro<br>• Combinations of miRNAs that were overexpressed in MS patients demonstrated a synergistic effect in reducing inducible Treg (iTreg) generation
Wang X et al. (52), 2019	Quasi-experimental study	SLE	27 SLE patients; 24 Active SLE, 3 Inactive SLE	21 Healthy Controls; Healthy CD4+ T cells	• HERV-E clone 4–1 mRNA expression was upregulated in CD4+ T cells from SLE patients, showing potential as a diagnostic biomarker<br>• DNA hypomethylation enhances direct activation of HERV-E clone 4–1 transcription by NFAT1 and ER-α

					• HERV-E clone 4–1 3′LTR acts as a miR-302d sponge, upregulating MBD2 and IRF9, leading to global DNA hypomethylation and increased IL-17
Wang Y et al. (51), 2017	Quasi-experimental study	Ankylosing spondylitis	41 AS Patients	36 Healthy Controls; Healthy CD4+ T cells, Untreated cells	• miRNA-199a-5p expression, and autophagy-related genes LC3, beclin1, and ATG5, were decreased in AS T cells, correlating with increased TNF-α, IL-17, and IL-23 levels<br>• miRNA-199a-5p expression negatively correlated with the Ankylosing Spondylitis Disease Activity Score (ASDAS) and modified Stoke Ankylosing Spondylitis Spinal Score (mSASSS)<br>• Directly targeted by miRNA-199a-5p, Rheb inhibition decreased phosphorylation of mTOR and induced autophagy
Rasmussen et al. (64), 2015	Quasi-experimental study	SLE	14 SLE Patients	12 Healthy Controls; Healthy CD4+ T Cells, Untreated Cells	• miR-410 expression levels were decreased in SLE T cells<br>• Overexpression of miR-410 significantly reduced the expression levels of IL-10<br>• miR-410 suppresses the transcription activity of STAT3 by binding directly to the 3 'UTR of STAT3 mRNA; silence of STAT3 down regulated IL-10 expression in CD3+ T cells
Wu et al. (49), 2018	Quasi-experimental study	Psoriasis	30 Psoriasis Patients; 6 Mice per group	Healthy CD4+ T cells, WT mice, Untreated cells	• miR-210, highly expressed in psoriasis, induces Th17 and Th1 cell differentiation, but inhibits Th2 differentiation by repressing STAT6 and LYN expression

	Experimental animal study				• miR-210 ablation in mice and miR-210 inhibition by antagomir-210 blocked immune imbalance and development of psoriasis-like inflammation<br>• TGF-β and IL-23 enhance miR-210 expression by inducing HIF-1α, which recruits P300 and promotes histone H3 acetylation in the miR-210 promoter region
